# Optical Force Monitoring in Polymeric Materials with a Coumarin‐Based Mechanophore

**DOI:** 10.1002/anie.202513283

**Published:** 2025-08-25

**Authors:** Yang Li, Jess M. Clough

**Affiliations:** ^1^ Adolphe Merkle Institute University of Fribourg Chemin des Verdiers 4 Fribourg 1700 Switzerland

**Keywords:** Dyes, Fluorescence, Mechanochemistry, Polymers, Sensing

## Abstract

Optical mechanophores offer a powerful platform for the real‐time, nondestructive detection of mechanically induced damage in polymers. However, the exploitation of mechanophores for fundamental investigations of deformation processes and the prediction of failure in technologically critical applications has been constrained by their responsivity to other stimuli, laborious synthesis, and limited control of their photophysical and mechanical properties. Here, we report a cyclobutane‐based mechanophore that activates a fluorescent coumarin dye through the application of mechanical stress in solution and in the solid state. We demonstrate the preparation of the mechanophore in two synthetic steps, its incorporation into linear and crosslinked polymers, and its thermal and photochemical stability, establishing the basis for a new class of mechanophores with tunable spectral and mechanical characteristics.

## Graphical abstract

Coumarin dyes are reacted with acrylates to form a mechanophore via an accessible two‐step synthesis that exhibits exclusive responsivity to force. Mechanical deformation of polymeric materials functionalized with the mechanophore activates blue fluorescence visible to the naked eye, enabling the optical detection of force‐induced molecular damage in the material.

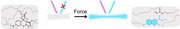

Advances in polymer mechanochemistry over the past two decades have enabled the design of polymeric materials with increasingly sophisticated responses to mechanical force.^[^ ^1^, ^2^, ^3^, ^4^ ^]^ In particular, stress‐responsive polymers that signal mechanical events by way of optical changes have emerged as valuable tools for the visualization and quantification of force and strain at different length scales,^[^ ^5^ ^]^ with potential applications ranging from materials damage detection^[^ ^6^ ^]^ and structural health monitoring^[^ ^7^ ^]^ to information encryption.^[^ ^8^ ^]^ To impart optical mechano‐responsivity to polymers, small quantities of force‐sensitive molecular units, known as “mechanophores”, can be covalently integrated into the polymer backbone.^[^ ^9^ ^]^ When subjected to stress, these mechanophores can undergo bond cleavage or structural rearrangements, resulting in the formation of colored,^[^ ^10^ ^]^ fluorescent^[^ ^11^ ^]^ or chemiluminescent^[^ ^12^, ^13^ ^]^ species that are readily detectable via standard imaging or spectroscopic techniques.^[^ ^6^, ^14^, ^15^, ^16^ ^]^ Compared to other mechanical sensing methods, such as laser speckle imaging,^[^ ^17^ ^]^ digital image correlation,^[^ ^18^ ^]^ photoelasticity^[^ ^19^ ^]^ and electrical measurements,^[^ ^20^ ^]^ mechanophore‐based systems provide insights into the molecular‐level consequences of the application of force to polymers, such as covalent bond rupture,^[^ ^21^ ^]^ and can be broadly applied to different polymer classes.

Fluorescent mechanophores offer desirable sensing characteristics for the study of fracture and failure mechanisms in polymers. Mechano‐activated fluorescence emission signals can be detected in situ or post mortem,^[^ ^22^ ^]^ with greater sensitivity than changes in absorption,^[^ ^23^ ^]^ with fluorescence spectroscopy or microscopy.^[^ ^6^, ^24^ ^]^ Yet, mechanical activation in *solid‐state* polymeric materials has been reported for only a handful of fluorescent mechanophores, which can also exhibit competing responsivities or require significant synthetic effort to prepare. The mechanochemically induced isomerization of spiropyran to fluorescent merocyanine has been demonstrated in a wide range of polymeric materials, spanning poly(methyl acrylate),^[^ ^4^ ^]^ poly(methyl methacrylate),^[^ ^25^ ^]^ polydimethylsiloxane,^[^ ^26^ ^]^ polyurethane,^[^ ^27^ ^]^ and acrylate networks.^[^ ^28^, ^29^ ^]^ Despite its broad implementation, the spiropyran mechanophore suffers from significant drawbacks, including the low fluorescence yield of the merocyanine isomer (*ϕ*_f_ < 0.02),^[^ ^23^, ^30^ ^]^ which limits detection sensitivity, the sensitivity of spiropyran to its chemical environment, and the ring‐closing reaction of merocyanine under the excitation light required to image its fluorescence. Activating at larger forces (∼nN),^[^ ^31^ ^]^ the rhodamine mechanophore^[^ ^32^, ^33^, ^34^, ^35^ ^]^ offers greater quantum yields,^[^ ^36^ ^]^ though can also exhibit photo‐^[^ ^33^, ^34^ ^]^ and acidochromism.^[^ ^35^ ^]^ Similarly, Diels–Alder adducts of anthracene and maleimide can be mechanically activated to produce fluorophores with high fluorescence quantum yields (*ϕ*_f_ = 0.72),^[^ ^23^, ^37^ ^]^ and recent work has started to engineer these motifs for specific spectral and mechanical properties, but achieving this control remains challenging.^[^ ^21^, ^38^, ^39^, ^40^ ^]^ Alongside covalent mechanophores, molecular probes that undergo force‐induced conformational changes at small forces (∼10–100 pN) have made it possible to detect small, elastic strains in polymeric matrices and to map these strains at the microscopic length scale.^[^ ^6^, ^41^ ^]^ These systems are, however, typically restricted to in situ sensing, and their spectral responses can be difficult to interpret. For example, flapping mechanophores based on the cyclooctatetraene anthracene dimer are sensitive to free volume.^[^ ^42^, ^43^ ^]^ Thiophene‐quinoxaline‐thiophene torsional springs offer a greyscale response to mechanical force, albeit in rigid polymeric matrices and over a narrow spectral range.^[^ ^44^ ^]^ Fluorescent mechanophores that rely on supramolecular interactions between aromatic dyes can also exhibit inter‐mechanophore interactions, complicating the interpretation of their mechanical responses.^[^ ^6^, ^41^ ^]^ One way to prevent this is by incorporating the dyes in mechanically interlocked architectures, though this approach requires significant synthetic effort.^[^ ^45^, ^46^ ^]^

The cyclobutane scaffold is a popular mechano‐responsive motif that offers potentially attractive features for fluorescent mechano‐sensing.^[^ ^47^ ^]^ Readily prepared via [2+2] photoaddition^[^ ^48^ ^]^ or the polyaddition of cyclobutene,^[^ ^49^ ^]^ the cyclobutane unit is thermally stable^[^ ^50^, ^51^, ^52^ ^]^ (the cycloreversion being thermally forbidden) and optically transparent at wavelengths longer than 300 nm.^[^ ^48^ ^]^ By contrast, cyclobutanes can readily undergo cycloreversion under force, yielding two alkenes through a diradical intermediate.^[^ ^53^ ^]^ Moreover, the mechanically generated alkenes can form conjugated structures, notably polyacetylene from ladderanes,^[^ ^54^ ^]^ cinnamoyl groups from cinnamate dimers,^[^ ^55^, ^56^, ^57^ ^]^ and coumarins from coumarin dimers.^[^ ^58^, ^59^ ^]^ Given the highly desirable properties of coumarins for fluorescent sensing,^[^ ^60^ ^]^ including good fluorescence quantum yields^[^ ^61^, ^62^, ^63^, ^64^ ^]^ and tunable spectral properties,^[^ ^65^ ^]^ their mechanochemical activation from cyclobutane mechanophores has received curiously little attention, especially in solid‐state polymeric materials. Head‐to‐head and head‐to‐tail coumarin dimers were reported to activate in silica‐filled styrene‐butadiene rubber subjected to compression at −15 °C, but the presence of highly absorbing catalyst impurities in the polymeric material prevented optical characterization of the material in the solid state, necessitating chemical degradation of the matrix to detect the coumarin products.^[^ ^66^ ^]^ The limited deployment of coumarin dimer mechanophores for solid‐state damage detection in polymers may be explained by the somewhat low fluorescence quantum yields of the coumarin derivatives used in these studies^[^ ^67^ ^]^ and possibly also by the relatively high forces required for their dissociation, which was cited as the reason for the poor selectivity in activation of a head‐to‐head coumarin dimer with respect to nonspecific chain scission in solution‐state sonication experiments.^[^ ^68^ ^]^ Activation force has been observed to influence mechanophore activation in solid‐state polymeric materials,^[^ ^69^ ^]^ though more systematic studies are required to understand the impact of mechanophore selectivity as a design parameter in this context, and how mechanophores could be applied to study the broad force distributions at the molecular level in solid‐state polymers.^[^ ^70^, ^71^, ^72^, ^73^ ^]^ In general, coumarin‐based motifs remain greatly under‐exploited for fluorescent force‐reporting, in spite of the recent advances in the design of cyclobutane mechanophores for improved mechanotransduction.^[^ ^74^, ^75^, ^76^, ^77^, ^78^ ^]^

Overcoming these limitations, we report a novel mechanophore based on a [2+2] adduct of coumarin and acrylate (Figure 1). Prepared in a two‐step synthesis, the adduct is thermally and photochemically stable, and readily incorporated into linear poly(methyl acrylate) (PMA) as well as crosslinked PMA networks. The mechanically induced cycloreversion produces a bright blue fluorescence visible to the naked eye, which is demonstrated in solution by sonication and in the solid state by tensile stretching, and the mechano‐generated coumarins are characterized by fluorescence and absorbance spectroscopy. Computational modeling based on the constrained geometries simulate external force (CoGEF) method, confirms the experimentally observed mechanochemical reactivity and allows us to rationalize the improvement in mechanical sensitivity with respect to existing mechanophores. The photoaddition reaction to form the adduct is mediated by visible light and has a broad substrate scope, which we expect will enable the preparation of a new class of mechano‐responsive cyclobutane motifs that activate coumarins exhibiting a wide range of spectral properties.

**Figure 1:**
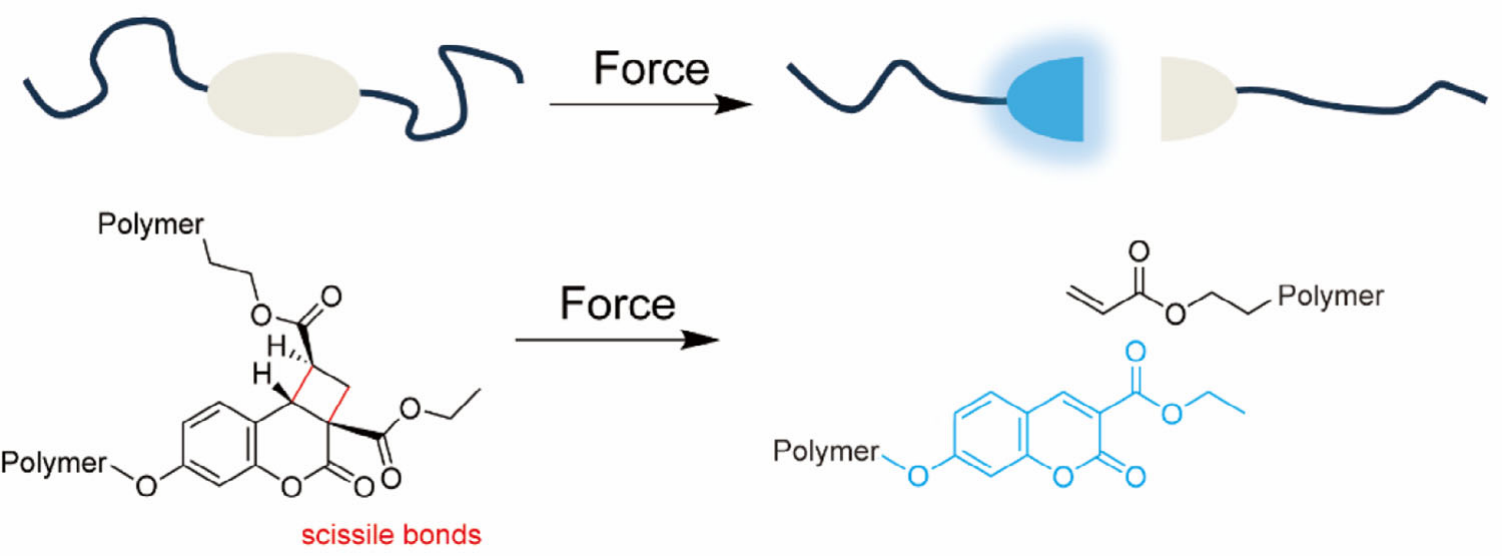
Schematic depiction of the mechanochemical activation of fluorescent coumarin and an acrylate from a nonfluorescent coumarin‐acrylate adduct.

Density functional theory (DFT) calculations based on the CoGEF method were first performed to evaluate the potential mechanochemical reactivity of the coumarin‐acrylate adduct.^[^ ^31^, ^79^ ^]^ Progressive elongation of both exo‐ and endo‐isomers of the adduct was simulated by constraining the distance between the two terminal atoms representing the polymer attachment points and performing a series of geometry optimizations, which in both cases led to the desired retro‐[2+2] cycloaddition reaction and generation of the coumarin structure. The maximum energy of this transformation, *E*_max_, for the exo‐ and endo‐diastereomers was 419 and 325 kJ mol^−1^, respectively, and the predicted rupture force, *F*_max_, 2.93 and 3.55 nN, respectively (Figures 2 and S1), with the latter metric considered the more reliable indicator of relative mechanochemical activity.^[^ ^31^ ^]^ It is noteworthy that these values are considerably smaller than those previously reported for mechanically activated coumarin dimers (633–1017 kJ mol^−1^, *F*_max_ = 5.6–5.9 nN),^[^ ^31^ ^]^ indicating that the coumarin‐acrylate adduct may offer greater mechanochemical reactivity.^[^ ^80^ ^]^ Interestingly, the exo‐adduct underwent stepwise decomposition, suggesting a biradical intermediate, while the scissile bonds in the endo‐adduct broke in a concerted fashion, which could result from differing steric interactions between the pendant groups of the cyclobutane,^[^ ^77^ ^]^ although we note that caution is required in drawing mechanistic conclusions from CoGEF results.^[^ ^31^ ^]^ Control calculations were also conducted on four coumarin‐acrylate adducts that were expected to display minimal or reduced mechanochemical reactivity (with the attachment points connected to the C1 and C2 positions, or the C1 and C4 positions of the cyclobutane, Figures S2–S5). Three of the four adducts did not undergo the retro‐[2+2] cycloaddition reaction, and ester C─O bonds peripheral to the cyclobutane broke instead at *F*_max_ of ca. 6 nN (Figures S2, S3, and S5), while the remaining adduct underwent cleavage of the cyclobutane bonds in a flex‐type activation at a large force (4.5 nN) (Figure S4). Based on these calculations, we focused the rest of our investigations on the exo‐adduct, which has a lower *F*_max_ than the endo‐adduct.

**Figure 2:**
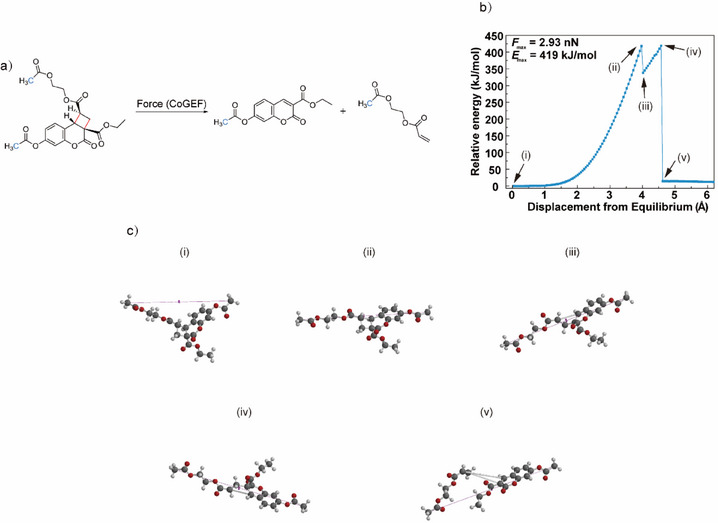
DFT calculations using the CoGEF method at the B3LYP/6–31G* level of theory for the mechanical elongation of the exo coumarin‐acrylate adduct. a) Schematic of the mechanically induced retro‐[2+2] cycloaddition of the adduct to form coumarin and acrylate, showing the pulling points in blue and scissile bonds in red. b) Relative energy plotted as a function of displacement from equilibrium. c) Computed structures at points in the CoGEF calculation indicated in b): i) equilibrium geometry, ii) immediately prior to first bond cleavage, iii) immediately after first bond cleavage, iv) immediately prior to second bond cleavage, and v) immediately after second bond cleavage.

With these preliminary indications of the desired mechanochemical reactivity from the CoGEF calculations, the hypothesized mechanophore and polymeric materials incorporating the new probe were prepared by straightforward synthetic steps (Scheme 1). The cyclobutane scaffold was synthesized by a photocatalytic cycloaddition reaction between hydroxycoumarin (**OH‐CM**) and hydroxyethyl acrylate (**HEA**) under visible light irradiation in the presence of bis(3,5‐difluoro‐2‐(pyridin‐2‐yl)phenyl)(picolinoyloxy)iridium (FirPic) (Scheme 1).^[^ ^81^ ^]^ The resulting dihydroxy‐functionalized exo‐adduct of coumarin and hydroxyethyl acrylate (**OH‐CM‐HEA**) was isolated from the diastereomeric mixture of exo‐ and endo‐adducts by column chromatography, and subsequently converted by esterification into two derivatives: the atom transfer radical polymerization (ATRP) initiator **CM‐HEA‐I** and the bis(methacrylate) crosslinker **CM‐HEA‐X**, which enabled the incorporation of the probe in linear poly(methyl acrylate) (**PMA‐C**) and crosslinked poly(methyl acrylate) networks (**PMA‐N**), respectively. The structures of the synthesized compounds were confirmed through ^1^H and ^13^C NMR and HR‐MS spectroscopy (Figures S20–S32). The thermal and photochemical stability of the coumarin‐acrylate adduct was established in a series of control experiments on the diol‐functionalized derivative, **OH‐CM‐HEA** (Text S1 and Figures S6, S8 and S9), and thermogravimetric analysis and differential scanning calorimetry confirmed that the diol‐functionalized adduct **OH‐CM‐HEA** remained stable up to approximately 220 °C (Figures S8, S9). The adduct was therefore not expected to undergo competing thermal or photochemical decomposition in the subsequent investigations of its mechanical reactivity.

**Scheme 1:**
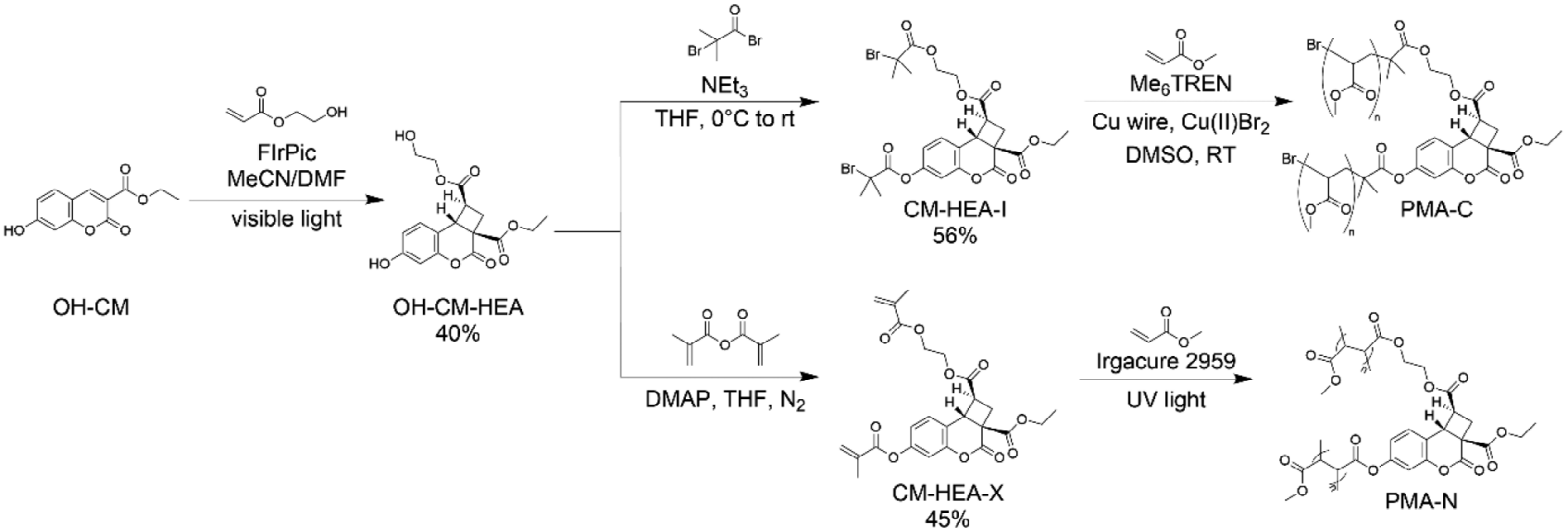
Synthetic preparation of coumarin‐acrylate adduct initiator for ATRP (**CM‐HEA‐I**) and crosslinker (**CM‐HEA‐X**), and linear and crosslinked poly(methyl acrylate) containing the adduct (**PMA‐C** and **PMA‐N**, respectively).

To investigate its response to mechanical force, **CM‐HEA** adducts were first incorporated into linear poly(methyl acrylate) (PMA). Two mechanophore‐centered PMA polymers were prepared with weight‐average molecular weights (M_w_) of 128 kDa (**PMA‐C‐128k**, *Đ* = 1.12) and 14 kDa (**PMA‐C‐14k**, *Đ* = 1.09) by controlled radical polymerization of methyl acrylate (MA) with copper wire and tris 2‐(dimethylamino)ethyl amine (Me_6_TREN) in DMSO under a nitrogen atmosphere, and characterized by ^1^H NMR spectroscopy (Figure 3) and size exclusion chromatography (SEC) (Figure 4a,b). Two peaks were observed in the refractive index trace of **PMA‐C‐128k** at retention times of 12.1 and 12.9 min (ratio of areas = 4.3:1; Figure 4a), with the lower MW fraction attributed to PMA end‐functionalized with the adduct resulting from mono‐initiation of the **CM‐HEA‐I** initiator (Text S2).^[^ ^82^ ^]^ Being mechanochemically inactive, the presence of these end‐functionalized polymers was not expected to influence the results of the following investigations on **PMA‐C** (aside from limiting the apparent mechanochemical conversion).

**Figure 3:**
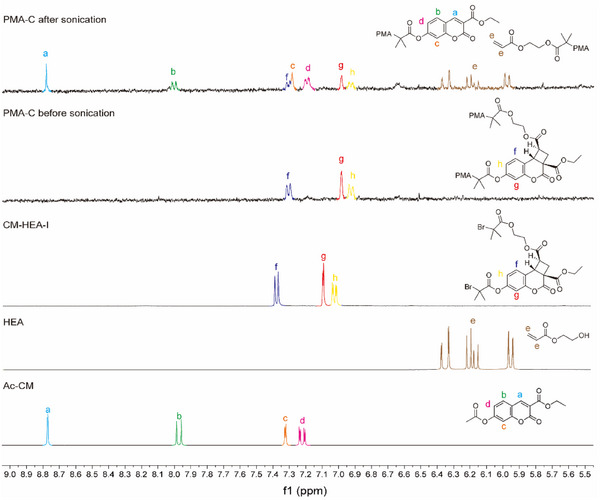
^1^H NMR spectra in d_6_‐DMSO showing the activation of coumarin by the mechanically induced [2+2] retro‐cycloaddition of the coumarin‐acrylate adduct mechanophore in **PMA‐C** upon ultrasound sonication in acetonitrile solution. From bottom to top: **Ac‐CM**, **HEA**, **CM‐HEA‐I**, **PMA‐C‐128k** before sonication, and **PMA‐C‐128k** after 80 min sonication. The spectrum of **PMA‐C‐128k** after sonication shows a mixture of the starting adduct, and the coumarin and acrylate products of the retro‐cycloaddition.

**Figure 4:**
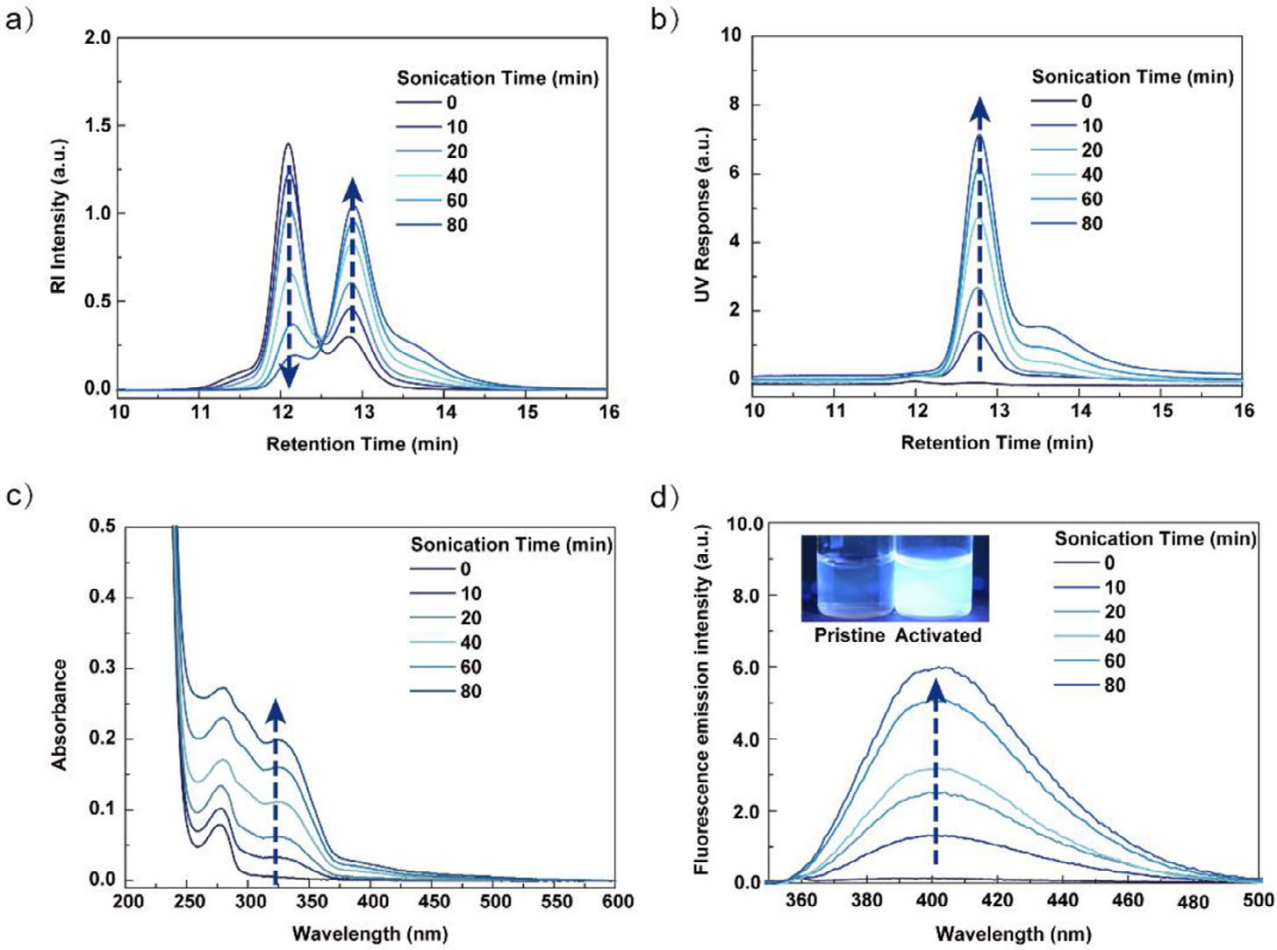
Mechanical activation of **PMA‐C‐128k** by ultrasound sonication in dilute solution: a) SEC‐RI traces, b) SEC‐UV (346 nm) chromatograms, c) UV–vis absorption spectra, and d) fluorescence emission spectra. Inset photographs in d) show the fluorescence change of the sonicated **PMA‐C‐128k** solution before (left) and after (right) sonication under irradiation at *λ*_ex_ = 365 nm. All spectra recorded at a concentration of 2.5 mg mL^−1^ in acetonitrile.

To enable their mechanical activation, **PMA‐C‐128k** and **PMA‐C‐14k** were subjected to ultrasound sonication in dilute solutions (acetonitrile, 5 mg mL^−1^, 0 °C). Sonication of polymer solutions initiates cavitation, where bubbles nucleate, grow, and collapse, generating shear forces that pull nearby polymer chain ends into the imploding void. When the molecular weight of the polymer chain is above a certain threshold (ca. 20 kDa for cyclobutane mechanophores in PMA),^[^ ^83^ ^]^ the forces generated along the main chain can lead to irreversible covalent bond rupture.^[^ ^9^ ^]^ Characterization of the polymer solutions by ^1^H NMR spectroscopy permitted identification of the chemical species produced upon mechanical activation. In the spectra of **PMA‐C‐128k** after sonication, we observed peaks in the range of 6.90–8.80 ppm corresponding to the coumarin (Figure 3, blue, green, and pink) and a series of peaks from 5.90–6.40 ppm (brown) corresponding to the acrylate in **HEA**, indicating that the cyclobutane can undergo the desired mechanically induced transformation to produce coumarin and acrylate. By contrast, the ^1^H NMR spectra of low MW **PMA‐C‐14k** before and after sonication showed no significant changes in the proton resonances of the adduct (Figure S10). Sonication of small molecule **CM‐HEA‐I** in the presence of a high MW PMA (150 kDa) also did not lead to cleavage of the adduct, as confirmed by ^1^H NMR spectroscopy (Figure S12).

Size exclusion chromatography (SEC) of the solutions of **PMA‐C‐128k** revealed the changes in the molecular weight distribution and in the optical properties upon sonication (Figure 4). As sonication progressed from 0 to 80 min, chain scission of **PMA‐C‐128k** resulted in the generation of daughter fragments with approximately half the starting molecular weight, leading to a decrease in the intensity of the peak at shorter retention times (12.1 min, 141 kDa) and an increase at longer retention times (12.9 min, 71 kDa) (Figure 4a). The emergence of a low MW shoulder at a retention time of ca. 13.6 min likely indicated secondary fragmentation of daughter chains by nonspecific chain scission. In addition, the UV chromatograms revealed the production of a chromophore absorbing at 346 nm during the sonication process (Figure 4b), which was associated predominantly with the low MW species (12.9 and 13.6 min). By contrast, low MW **PMA‐C‐14k** exhibited only a slight increase in UV intensity SEC‐UV chromatograms at low MWs (14.7 min), which likely resulted from the mechanochemical activation of the small fraction of PMA‐C species in the high MW shoulder of the initial RI profile (Figure S11).

UV–vis and fluorescence spectroscopy of the sonicated polymer solutions permitted further elucidation of the optical properties of the coumarin product and in situ monitoring of its mechanical activation over time. The appearance of a new peak at 325 nm in the UV–vis spectra of **PMA‐C‐128k** after sonication could be attributed to the generated coumarin fragment (Figures 4c, S7 and S16). Moreover, blue fluorescence was clearly visible by eye under irradiation with UV light (*λ*_ex_ = 365 nm) in the **PMA‐C‐128k** solution sample that had been sonicated for 80 min (photograph, inset Figure 4d). This observation was confirmed by fluorescence spectroscopy, which showed the appearance of a new fluorescence peak matching that of the control coumarin (Figure S17) that increased significantly in intensity with sonication time (Figure 4d). These spectra were background‐corrected following the procedure of Robb et al. (Figure S13 and Text S4).^[^ ^84^ ^]^ By contrast, the fluorescence intensity of sonicated **PMA‐C‐14k** showed minimal change (Figure S14), which confirmed the lack of significant mechanochemical reactivity observed in the ^1^H NMR and SEC experiments. Similarly, no mechano‐activated fluorescence was observed from solutions of small molecule **CM‐HEA‐I** sonicated in the presence of a high MW PMA (150 kDa) (Figure S15). The mechanochemical conversion and selectivity of **CM‐HEA** were calculated from the UV–vis spectra after sonication for 80 min to be 73% and 68%, respectively (Figure S16 and Text S3).^[^ ^68^, ^85^ ^]^ The selectivity of **CM‐HEA** is considerably higher than the value of 32% reported for a coumarin dimer mechanophore (in PMA across a range of molecular weights).^[^ ^68^ ^]^

The results obtained in polymer solutions prompted us to advance our system to the solid state. 1 mm‐thick samples of poly(methyl acrylate) networks (**PMA‐N**) crosslinked by bis(methacrylate)‐functionalized mechanophores were prepared by photopolymerizing methyl acrylate (**MA**) and the crosslinker with Irgacure 2959 photoinitiator between glass slides separated by a spacer. Rectangular samples of **PMA‐N** subjected to a single uniaxial tensile test to failure at a strain rate of 10.5% s^−1^ exhibited a blue fluorescence emission under UV irradiation visible by eye (Figure 5, *λ*_ex_ = 365 nm, inset photographs). Fluorescence spectroscopy on the solid samples before and after tensile stretching showed the emergence of a prominent emission band with an intensity maximum at *λ*_em_ = 410 nm that matched the emission spectrum of small molecule coumarin **Ac‐CM** (Figure S17), confirming the mechanically induced formation of the coumarin dye. Control PMA networks crosslinked with poly(ethylene glycol) diacrylate, subjected to similar tensile loading cycles, were not observed to fluoresce. Compared to **PMA‐PEGDA**, **PMA‐N** exhibited slightly greater stresses (Figures S18 and S19), which could be attributed to differences in the crosslinker.^[^ ^86^ ^]^ To our knowledge, this is the first report of the fluorescence detection of mechanochemical coumarin activation from within a solid‐state polymeric material.

**Figure 5:**
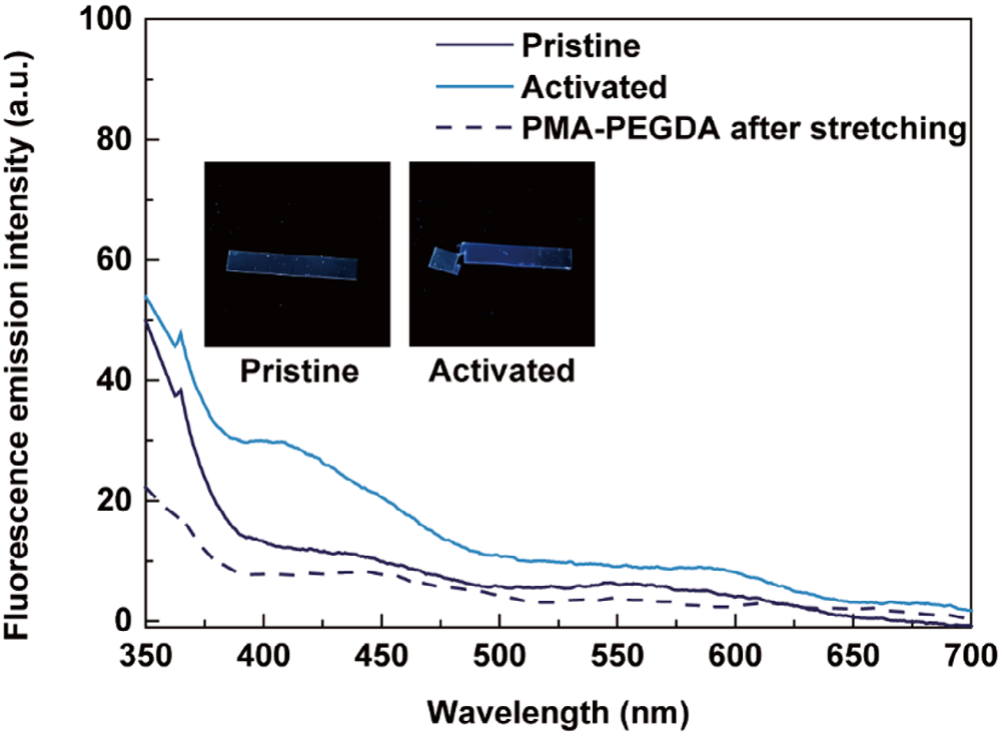
Fluorescence spectra of a **PMA‐N** film before and after tensile stretching, and of a PMA‐PEGDA reference sample after tensile stretching, recorded in an optical fiber set‐up. The photographs (inset) show the **PMA‐N** sample before and after stretching under irradiation at *λ*_ex_ = 365 nm.

In summary, we have demonstrated a new cyclobutane‐based mechanophore that can be mechanically activated in a retro‐[2+2] cycloaddition to activate a coumarin fluorophore. The application of mechanical stress to polymers containing the new mechanophore in both the solution and solid state generated coumarins that were characterized by SEC, UV–vis absorption, fluorescence, and ^1^H NMR spectroscopy. Moreover, the preparation of this motif class uses a visible‐light‐driven protocol, which will enable access to a broad range of coumarin‐based mechanophores with optical properties that span the visible spectrum and readily tunable mechanochemical reactivity. In the future, we expect that the accessible design of this mechano‐responsive motif will facilitate its integration into advanced sensing and imaging platforms, which, for example, exhibit orthogonal responsivities to light and force.

## Conflict of Interests

The authors declare no conflict of interest.

## Supporting information



Supporting Information

## Data Availability

The data that support the findings of this study are available in the Supporting Information of this article and for download at the Zenodo repository at https://zenodo.org/records/16890533.
