# Supplementary material for: Optical Force Monitoring in Polymeric Materials with a Coumarin‐Based Mechanophore
Source: Angew Chem Int Ed Engl. 2025 Aug 25;64(41):e202513283. doi: 10.1002/anie.202513283 (PMC12501738; doi:10.1002/anie.202513283)
Supplement: Supplementary file 1 — Supporting Information [file ANIE-64-e202513283-s001.docx]

**Supporting Information**

# Optical Force Monitoring in Polymeric Materials with a Coumarin-based Mechanophore

Yang Li,^a^ Jess M. Clough*^a^

^a^ Adolphe Merkle Institute, University of Fribourg, Chemin des Verdiers 4, 1700, Fribourg, Switzerland

E-mail: [jessica.clough@unifr.ch](mailto:jessica.clough@unifr.ch)

**Table of Contents**

[Materials and Methods S1](#_Toc200231145)

[Synthetic Procedures S4](#_Toc200231146)

[Supplementary Figures S8](#_Toc200231147)

[NMR Spectra S21](#_Toc200231148)

[References S27](#_Toc200231149)

# Materials and Methods

**Materials**

Chemicals were purchased from Sigma Aldrich, Tokyo Chemical Industry Co. Ltd. (TCI), or Combi-Blocks and used without further purification unless otherwise stated. Solvents were purchased from ACROS and VWR. Methyl acrylate was purified by passing through a short column of neutral Al_2_O_3_ prior to use to remove the inhibitor. Deuterated solvents were purchased from Cambridge Isotope Laboratories, Inc.

**Nuclear magnetic resonance (NMR) spectroscopy**

NMR spectroscopy was carried out at 297.2 K on a Bruker Avance DPX 400 spectrometer at frequencies of 400.19 MHz for ^1^H nuclei and 100.63 MHz for ^13^C nuclei. Spectra were calibrated to the residual solvent peak of CDCl_3_ (7.26 ppm ^1^H NMR; 77.16 ppm ^13^C NMR), DMSO-d_6_ (2.50 ppm ^1^H NMR; 39.52 ppm ^13^C NMR), or acetonitrile-d_3_ (1.94 ppm, ^1^H NMR; 1.32 ppm, 118.26 ppm ^13^C NMR). Data were evaluated with the MestReNova software suite (V 11.0) and all chemical shifts (δ) are reported in parts per million (ppm) relative to tetramethylsilane with coupling constant in Hz (multiplicity: s = singlet, d = doublet, dd = double doublet, t = triplet, m = multiplet, br = broad signal).

**Mass spectrometry (MS)**

Mass spectrometry was carried out as service measurements by the Analytical Service at the University of Zurich. High-resolution electrospray mass spectra (HR-ESI-MS) were recorded on a *timsTOF Pro* TIMS-QTOF-MS instrument (*Bruker Daltonics* GmbH, Bremen, Germany). The samples were dissolved in MeOH at a concentration of ca. 50 µg ml^-1^ and analyzed via continuous flow injection (2 µL min^-1^). The mass spectrometer was operated in the positive (or negative) electrospray ionization mode at 4 000 V (-4 000 V) capillary voltage and -500 V (500 V) endplate offset with a N_2_ nebulizer pressure of 0.4 bar and a dry gas flow of 4 L min^-1^ at 180°C. Mass spectra were acquired in a mass range from *m/z* 50 to 2 000 at ca. 20 000 resolution (*m/z* 622) and at 1.0 Hz rate. The mass analyzer was calibrated between *m/z* 118 and 2 721 using an *Agilent* ESI-L low concentration tuning mix solution (*Agilent*, USA) at a resolution of 20 000 giving a mass accuracy below 2 ppm. All solvents used were purchased in LC-MS quality.

**Ultrasonication**

Sonication experiments were conducted using a Branson Digital Sonifier 450 equipped with a tapered microtip (1/8” / 3 mm diameter). Polymer solutions in MeCN (5 mg/mL) were introduced to a 25 mL Suslick cell and purged with nitrogen for 30 minutes. The distance between the tip and the bottom of the Suslick cell was 1 cm. Temperature control was facilitated by immersing the Suslick cell in an ice bath. The sonication probe was set at 20 % amplitude. Solutions were sonicated with a pulsed protocol (1.0 s on, 1.0 s off). The total time for experiments was 160 min (80 min effective sonication time).

**UV-vis absorption spectroscopy**

UV-vis absorption spectra were recorded on a Shimadzu UV-2401PC spectrophotometer using quartz cuvettes of 1 cm path length, and data were analyzed with the Spectra Manager software suite.

**Fluorescence spectroscopy**

Fluorescence spectroscopy in solution was carried out with a Horiba Fluorolog 3 spectrometer with right-angle illumination equipped with a 450 W Xenon light source for excitation and a FL-1030-UP photomultiplier as detector. Unless indicated otherwise, spectra were recorded with an excitation wavelength (λ_ex_) of 325 nm and 1.00 nm side entrance slit. Fluorescence spectra of the PMA and CM-HEA-I mixtures were measured using a PerkinElmer LS-50B Luminescence Spectrophotometer. Photoluminescence spectra of solid-state samples were acquired on an Ocean Optics USB4000-FL spectrometer, with an Ocean Optics LS-310 LED light source at an excitation wavelength of 310 nm and an Ocean Optics QR230-7-XRS SMA 905 optical fiber. The spectra were acquired by measuring films that were placed on a black piece of paper, with the optical fiber positioned at a distance of ca. 2 mm from the sample. The spectra were processed with a Savitsky-Golay filter in Origin (Points of Windows = 50, Polynomial Order = 1).

**Size exclusion chromatography (SEC)**

SEC analyses were performed on an Agilent Technologies 1200 series HPLC system equipped with an Agilent PLgel mixed guard column (particle size = 5 μm) and two Agilent PLgel mixed-D columns (ID = 7.5 mm, L = 300 mm, particle size = 5 μm). THF was used as eluent at a flow rate of 1.0 mL min^–1^ and a UV detector (Agilent 1200 series, λ = 346 nm), along with Wyatt Technology Corp. Optilab REX interferometric refractometer and miniDawn TREOS light scattering detector were used to monitor the signal. Data were processed with Agilent software and molecular weights (Mn) and polydispersity index (Đ) were determined based on poly(methyl methacrylate) standards.

**Thermogravimetric analyses (TGA)**

TGA was performed with a Mettler-Toledo TGA/DSC 1 Stare System. The temperature was increased from 25 °C to 500 °C at a heating rate of 10 °C min^-1^. The TGA results were analyzed using the STARe Evaluation software.

**Differential scanning calorimetry (DSC)**

DSC was conducted on a Mettler Toledo DSC 5+ STAR system under nitrogen up to 200 °C with a heating and cooling rate of 10 °C/min.

**Photochemical and thermal decomposition reactions**

A Dr. Hönle bluepoint 4 lamp equipped with a 390-500 nm filter (2.6 W cm^-2^) was used to prepare coumarin-acrylate adduct, **OH-CM-HEA**.

To check the thermal stability of the adduct, the NMR tube containing **OH-CM-HEA** in DMSO-d_6_ was put into an oil bath at 80 °C. To check the photostability of **OH-CM-HEA**, the Dr. Hönle bluepoint 4 lamp equipped with a 390 - 500 nm filter (2.6 W cm^-2^) and 320 - 390 nm filter (3.2 W cm^-2^), and a UVGL-58 handheld UV Lamp with a wavelength of 254 nm were used to irradiate an NMR tube containing a solution of **OH-CM-HEA**. The distance between the lamp and the NMR tube was 1 cm.

A Dr. Hönle LED Cube 100 UV chamber, operating at a wavelength of 365 nm and 178 mW cm^-2^ intensity, was used to carry out the UV-initiated free-radical polymerization reactions to prepare **PMA-N**. The curing time was 500 s for all samples.

**Tensile tests**

Tensile measurements were carried out according to ASTM D882, at room temperature, with a static material testing machine from Zwick/Roell equipped with a 200 N Xforce HP load cell. Rectangular-shaped samples (width 5.35 mm, thickness 1.1 mm) were measured at a strain rate of 10.5 % s^-1^.

**Photographs**

Photographs of samples were recorded with NIKON D7100, aperture (f/4), exposure time (1/50 s – 1/30 s), and ISO 4000 under irradiation with a UV hand lamp (λ_ex_ = 365 nm).

**DFT calculations**

CoGEF calculations were performed using Spartan’20 Parallel Suite according to previously reported methods.^[1,2]^ Ground state energies were calculated using DFT at the B3LYP/6-31G* level of theory in vacuum. Starting from the equilibrium geometry of the unconstrained molecule (relative energy = 0 kJ/mol), the distance between the terminal anchor atoms of the truncated structure was increased in increments of 0.05 Å and the energy was minimized at each step. This operation was carried out automatically using the Energy Profile calculation in Spartan. Calculations were run until a chemical transformation was predicted to occur, as evidenced by the rupture and reorganization of one or more covalent bonds. The maximum number of geometry optimization cycles increased beyond the default value using the GEOMETRYCYCLE option to ensure convergence at each step in the CoGEF profile.

The maximum force (*F*_max_) for each mechanochemical transformation was obtained from the slope between adjacent points on the energy–displacement curve. *F*_max_ is calculated using the slope between the two preceding data points at the displacement just before a discontinuity in the energy profile. These slope values were divided by Avogadro’s number and converted to units of nJ/m (equivalent to nN). The maximum energy (*E*_max_) is the highest energy relative to the relaxed, unconstrained molecule. It is extracted from the CoGEF curve at the displacement corresponding to *F*_max_, representing the peak of the energy profile.

# Synthetic Procedures

**
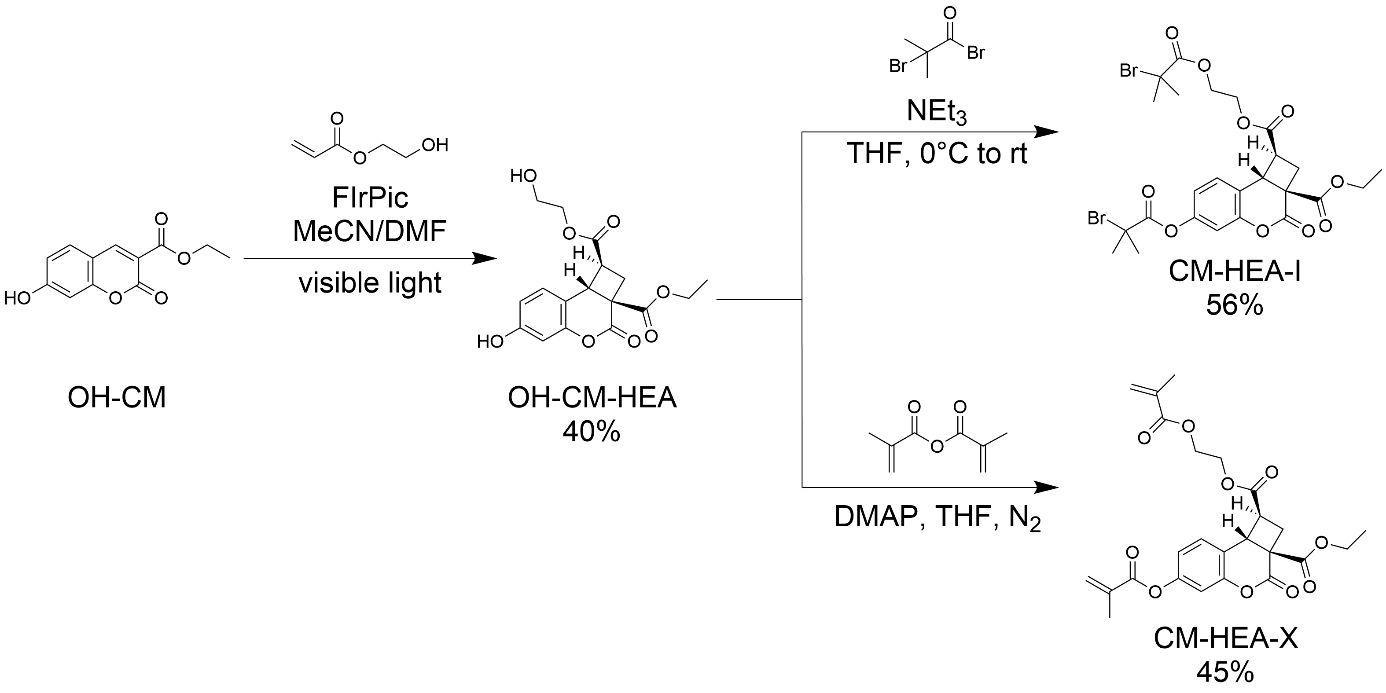
**

**Scheme S1.** Synthesis of coumarin-acrylate adduct.

Synthesis of **OH-CM-HEA**:

The procedure was adapted from literature.^[3]^ In a 20 mL snap-cap vial equipped with a magnetic stirring bar and fitted septum, **OH-CM** (2.10 g, 9 mmol), 2-hydroxyethyl acrylate (1.25 g, 10.8 mmol), and FIrPic (18.7mg, 0.027 mmol) were dissolved in CH_3_CN (9 mL) and DMF (2 mL). The mixture was bubbled with a stream of N_2_ for 30 min using a syringe needle. The vial was then irradiated for 15 h by using a Dr. Hönle bluepoint 4 lamp equipped with a 390-500 nm filter (2.6 W cm^-2^). After completion of the reaction, the solvent was removed under reduced pressure, and the residue was purified by flash column chromatography (SiO_2_) using n-hexane/EtOAc/AcOH (3:1:0.003 v/v/v) as eluent to a colorless liquid product **OH-CM-HEA** (1.26 g, 40 %). ^1^H-NMR (400 MHz, Acetonitrile-d_3_) δ 7.62 (s, 1H), 7.12 (d, J = 8.3 Hz, 1H), 6.66 (dd, J = 8.3, 2.4 Hz, 1H), 6.58 (d, J = 2.4 Hz, 1H), 4.27 – 4.11 (m, 4H), 4.03 (d, J = 8.7 Hz, 1H), 3.71 (t, J = 4.9 Hz, 2H), 3.31 – 3.09 (m, 2H), 3.00 (s, 1H), 2.74 – 2.64 (m, 1H), 1.20 (dt, J = 23.8, 7.1 Hz, 3H). ^13^C-NMR (101 MHz, Acetonitrile-d_3_) δ 172.72, 168.96, 167.19, 158.56, 152.73, 129.80, 112.92, 112.35, 104.18, 66.94, 62.92, 60.33, 48.16, 43.61, 42.32, 31.73, 13.79. HRMS (ESI): calcd. for C_17_H_18_NaO_8_: 373.0899 ([M+Na]+); found 373.0888.

Synthesis of **CM-HEA-I**:

The procedure was adapted from literature.^[4]^ An oven-dried round bottom flask equipped with a stir bar was charged with **OH-CM-HEA** (0.525 g, 1.5 mmol), and then THF (80 mL) was added. The mixture was bubbled with a stream of N_2_ for 30 min using a syringe needle. The solution was cooled to 0°C in an ice bath, followed by the dropwise addition of triethylamine (2.16 mL, 15.4 mmol) and α-bromoisobutyryl bromide (0.92 mL, 7.7 mmol) via syringe. The reaction mixture was allowed to warm to room temperature and stirred for 12 h. The mixture was extracted with ethyl acetate and washed with H_2_O (200 mL*3), dried with MgSO_4_, filtered and concentrated. The crude products were purified by flash column chromatography (SiO_2_) using n-hexane/EtOAc (3:1 v/v) as eluent to afford the desired product **CM-HEA-I** as a colorless liquid (0.55 g, 0.8 mmol, 56% yield). ^1^H-NMR (400 MHz, Acetonitrile-d_3_) δ 7.21 (d, J = 7.9 Hz, 2H), 6.85 (d, J = 1.9 Hz, 1H), 4.44 – 4.21 (m, 4H), 4.21 – 4.13 (m, 1H), 4.12 – 4.01 (m, 2H), 3.24 – 3.05 (m, 2H), 2.88 – 2.69 (m, 1H), 2.00 (s, 6H), 1.87 (s, 6H), 1.15 (t, J = 7.1 Hz, 3H).^13^C NMR-(101 MHz, Acetonitrile-d_3_) δ 171.47, 171.39, 169.88, 167.86, 165.76, 151.84, 151.23, 128.93, 117.96, 117.95, 110.68, 63.36, 62.74, 62.64, 55.30, 54.97, 47.32, 42.88, 41.72, 32.37, 30.65, 30.64, 30.57, 30.51, 24.00, 13.93.

Synthesis of **CM-HEA-X**:

The procedure was adapted from the literature.^[4]^ Compound **OH-CM-HEA** (0.4725 g, 1.35 mmol) and 4-dimethylaminopyridine (DMAP) (0.3 g, 2.425mmol) were dissolved in THF (15 mL). The mixture was bubbled with a stream of N_2_ for 30 min using a syringe needle. Then, methacrylic anhydride (0.65 mL, 3.5 mmol) was added. After stirring for 4 h, the organic phase was washed with H_2_O (30 mL), EtOAc (30 mL), 1 N HCl (20 mL) and 1 N NaHCO_3_ (20 mL), dried with anhydrous MgSO_4_, filtered and concentrated. The crude products were purified by flash column chromatography (SiO_2_) using n-hexane/EtOAc (3:1 v/v) as eluent to afford the desired product **CM-HEA-X** as a colorless liquid (0.334 g, 0.68 mmol, 45% yield). ^1^H-NMR (400 MHz, Acetonitrile-d_3_) δ 7.36 – 7.23 (m, 1H), 7.04 – 6.87 (m, 2H), 6.33 (p, J = 1.0 Hz, 1H), 6.10 (dt, J = 2.0, 1.0 Hz, 1H), 5.87 (p, J = 1.5 Hz, 1H), 5.67 (p, J = 1.6 Hz, 1H), 4.52 – 4.30 (m, 4H), 4.27 – 4.03 (m, 3H), 3.35 (dt, J = 10.3, 9.0 Hz, 1H), 3.18 (dd, J = 12.1, 10.3 Hz, 1H), 2.74 (ddd, J = 12.1, 8.9, 1.0 Hz, 1H), 2.06 (dd, J = 1.6, 1.0 Hz, 3H), 1.94 (dd, J = 1.6, 1.0 Hz, 3H), 1.21 (dt, J = 20.9, 7.1 Hz, 3H). ^13^C-NMR (101 MHz, Acetonitrile-d_3_) δ 172.40, 168.84, 167.61, 166.69, 166.22, 152.48, 152.31, 137.12, 136.56, 129.79, 128.07, 126.20, 119.40, 118.86, 111.64, 63.55, 63.26, 63.12, 48.00, 43.52, 41.90, 32.11, 18.22, 18.21, 13.99.

Synthesis of **Ac-CM**:

Literature synthetic procedures were followed.^[5]^ In a 25 mL two-necked flask equipped with a reflux condenser and a magnetic stir bar, **OH-CM** (0.28 g, 1.2 mmol) was added acetic anhydride (0.25 g, 2.4 mmol) and sodium thiosulfate pentahydrate (0.0297g, 0.12 mmol) in DMF (5 ml). The resulting solution was stirred at 70 ^o^C under a nitrogen atmosphere for 24 h. The mixture was extracted with EtOAc for three times and the combined organic layer was washed with saturated NaHCO_3_, dried over MgSO_4_, filtered and concentrated. The crude products were purified by flash column chromatography (SiO_2_) using n-hexane/EtOAc (1:1 v/v) as eluent to afford the desired product Ac-CM (72 mg, 50%) as a white solid. ^1^H-NMR (400 MHz, DMSO-d_6_) δ 8.78 (d, J = 0.7 Hz, 1H), 7.98 (d, J = 8.5 Hz, 1H), 7.37 – 7.29 (m, 1H), 7.23 (dd, J = 8.5, 2.2 Hz, 1H), 4.31 (q, J = 7.1 Hz, 2H), 2.33 (s, 3H), 1.32 (t, J = 7.1 Hz, 3H). ^13^C-NMR (101 MHz, DMSO-d_6_) δ 169.08, 162.99, 156.22, 155.74, 155.34, 148.79, 131.80, 119.59, 117.30, 116.19, 110.37, 61.71, 21.37, 14.55. HRMS (ESI): calcd. for C_14_H_13_O_6_: 277.0712 ([M+H]+); found 277.0701.

**Polymer synthesis.**

**
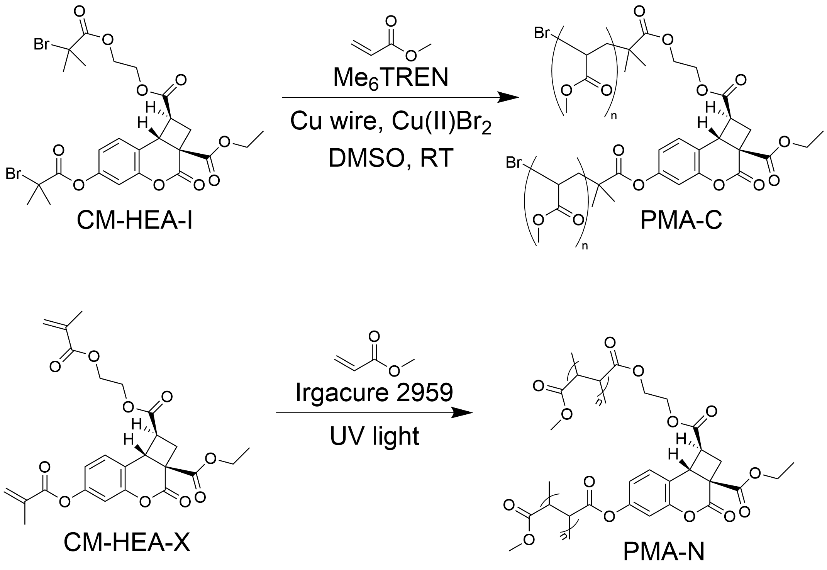
**

**Scheme S2.** Synthesis of polymers containing **CM-HEA** (**PMA-C** and **PMA-N**)

All polymers were prepared according to the procedure reported by Weder et al.,^[6]^ with modifications described below.

Synthesis of **PMA-C-128k**.

In a 20 mL snap-cap vial equipped with a magnetic stirring bar and fitted septum was charged with **CM-HEA-I** (32.4 mg, 0.05 mmol), Cu(0) (copper wire, length 9 cm, diameter 1 mm), methyl acrylate (6.30 mL, 35.0 mmol), CuBr_2_ (1.2 mg, 0.006 mmol) and dry DMSO (6.3 mL). The mixture was bubbled with nitrogen for 30 min, after which tris[2-(dimethylamino)ethyl]amine (0.0047 mL, 0.018 mmol) was added and the mixture was stirred for 1.5 h. The viscous mixture was diluted with THF and filtered over silica to remove copper. The solution was concentrated and then precipitated three times with cold methanol. The precipitate was collected, and the supernatant was discarded to give a white polymer. After 24 h drying in air at ambient temperature in a fume hood, it was transferred to an oven and dried under vacuum at 50 °C to obtain a white polymeric solid (1.50 g, 50% yield). ^1^H NMR (400 MHz, DMSO-d_6_) δ 3.71 – 3.47 (m, 3H), 2.39 – 1.40 (m, 3H). SEC (THF, PMMA): *M*_n_ = 114 900 g mol^–1^; *M*_w_ = 128 100 g mol^–1^, Ð = 1.12.

Synthesis of **PMA-C-14k**.

In a 20 mL snap-cap vial equipped with a magnetic stirring bar and fitted septum was charged with **CM-HEA-I** (32.4 mg, 0.05 mmol), Cu (0) (copper wire, length 9 cm, diameter 1 mm ), methyl acrylate (0.90 mL, 5.0 mmol), CuBr_2_ (1.2 mg, 0.003 mmol) and dry DMSO (2 mL). The mixture was bubbled with nitrogen for 30 min, after which tris[2-(dimethylamino)ethyl]amine (0.0047 mL, 0.018 mmol) was added and the mixture was stirred for 1.5 h. The viscous mixture was diluted with THF and filtered over silica to remove Cu. The solution was concentrated and then precipitated three times with cold methanol. The precipitate was collected, and the supernatant was discarded to give a white polymer. After 24 h drying in air at ambient temperature in a fume hood, it was transferred to an oven and dried under vacuum at 50°C to obtain a white polymeric solid (0.17 g, 41% yield). ^1^H NMR (400 MHz, DMSO-d_6_) δ 2.39 – 1.39 (m, 3H). SEC (THF, PMMA): *M*_n_ = 12 700 g mol^–1^; *M*_w_ = 13 900 g mol^–1^, Ð = 1.09.

Synthesis of crosslinked **PMA-N**.

Irgacure 2959 (1.5 mg, 0.00446 mmol) and **CM-HEA-X** (53.5 mg, 0.11 mmol) were placed in a vial with methyl acrylate (1 mL, 11 mmol). The mixture was sparged with N_2_ for 1 min. Two glass microscope slides were covered carefully with clear tape. Two pieces of parafilm were cut and folded and used as spacers to separate the glass slides. The slides were then clamped together with bulldog clips, and the solution was added to the polymerization cell with a Pasteur pipette. This was then immediately irradiated with 365 nm light for 500 s. The glass slides were separated and the polymer film was removed, then dried in air over 3 days.

Synthesis of crosslinked **PMA-PEGDA**.

Irgacure 2959 (1.5 mg, 0.00446 mmol) and **PEGDA** (average M_n_ 250, 22.0 mg, 0.11 mmol) were placed in a vial with methyl acrylate (1 mL, 11 mmol). The mixture was sparged with N_2_ for 1 min. Two glass microscope slides were covered carefully with clear tape. Two pieces of parafilm were cut and folded and used as spacers to separate the glass slides. The slides were then clamped together with bulldog clips, and the solution was added to the polymerization cell with a Pasteur pipette. This was then immediately irradiated with 365 nm light for 500 s. The glass slides were separated and the polymer film was removed, then dried in air over 3 days.

**Supplementary text S1: Thermal and photochemical stability of OH-CM-HEA**

^1^H NMR spectroscopy of CD_3_CN solutions of the adduct showed no significant changes following heating at 80 °C for 3 h, irradiation with 390 – 500 nm light for 30 min, irradiation with 320 – 390 nm light for 60 min and irradiation with 254 nm light for 120 min, with the exception of small changes in the chemical shifts of protons corresponding to the hydroxyl groups (from 3.00 ppm and 7.62 ppm to 3.55 ppm and 7.55 ppm, respectively) (Figure S6). It is also noted that the adduct is stable to the conditions of its synthesis (390 – 500 nm, 2.6 W cm^-2^, 15 h) and UV-initiated free radical polymerization (365 nm, 178 mW cm^-2^, 500 s) (Figure S6).

**Supplementary text S2: ^1^H NMR spectroscopy of PMA-C-128k before sonication.**

^1^H NMR spectroscopy confirmed that the bimodal molecular weight distribution was not the result of decomposition of the coumarin-acrylate adduct under ATRP conditions (Fig. 3). In particular, the ^1^H NMR spectrum of **PMA-C-128k** (before sonication) does not contain any characteristic resonances of the coumarin or of hydroxyethyl acrylate, observed in the spectra of 7-acetoxy-3-carbethoxycoumarin (**Ac-CM**) and hydroxyethyl acrylate **HEA**. Moreover, the aromatic peaks in the range of 6.90 – 7.35 ppm in the spectra of initiator **CM-HEA-I** and **PMA-C-128k** (before sonication) are similar, indicating that the core of the adduct remained intact (Fig. 3) and validating its successful integration into the polymer chain without significant decomposition. Based on these observations, the lower MW fraction in **PMA-C-128k** was attributed to PMA end-functionalized with the adduct, possibly resulting from mono-initiation of the **CM-HEA-I** initiator.^[7]^ Being mechanochemically inactive, the presence of these end-functionalized low MW polymers was not expected to influence the results of the following investigations on **PMA-C** (aside from limiting the apparent mechanochemical conversion).

**Supplementary text S3: Calculation of coumarin-acrylate adduct mechanophore activation and mechanochemical selectivity**

The final aliquots of **PMA-C** and MeCN (80 minutes effective sonication time) were transferred to a UV-Vis spectroscopy cuvette and their absorbance spectra were measured. By subtracting the absorbance value (325 nm) of MeCN ($A_{\mathrm{MeCN}}^{325 nm}$) from the corresponding **PMA-C** absorbance value ($A_{\mathrm{end}}^{325 nm}$), and dividing that by the absorbance of a 19.5 µM solution of Ac-CM ($A_{Ac-CM}^{325 nm}$, corresponding to the theoretical absorbance at 100% conversion), the percentage of activated **PMA-C** (k_1_), i.e., the mechanochemical conversion, could be determined (equation 1):^[8]^

$fraction of activated mechanophore= \frac{A_{\mathrm{end}}^{325 nm}-A_{\mathrm{MeCN}}^{325 nm}}{A_{Ac-CM}^{325 nm}}$ (1)

The fraction of total scission (k_2_) was calculated from SEC-RI from the starting *M*_n_ (*M*_n,start_) and the *M*_n_ after sonication (*M*_n,end_) of the polymer according to the equation 2, which assumes that each scission results in one new chain end:^[9]^

$fraction of total scission events= \frac{M_{n,end}^{-1}-M_{n,start}^{-1}}{M_{n,start}^{-1}}$ (2)

The selectivity, or selective scission, represents the percentage of the selectively cleaved mechanophore in a sample of n polymer chains at the desired position (yielding the coumarin fluorophore) out of the total number of scission events along the polymer chain in the same sample of n polymers. This scission selectivity (in %) was calculated as follows (equation 3):

$selective scission= \frac{k_{1}}{k_{2}}\times100\%$ (3)

**Supplementary text S4: Correction for background fluorescence**

Background fluorescence intensity was calibrated using the method described by Robb et al.^[10]^ To control for an increase in fluorescence intensity during ultrasonication resulting from the sonication of the solvent, a blank of acetonitrile was sonicated according to the sonication methods described above. The fluorescence intensity was found to increase linearly with sonication time. Fitting this time-dependent fluorescence response to a line provided a background correction factor that was subtracted from each fluorescence measurement (Figure S10).

# Supplementary Figures


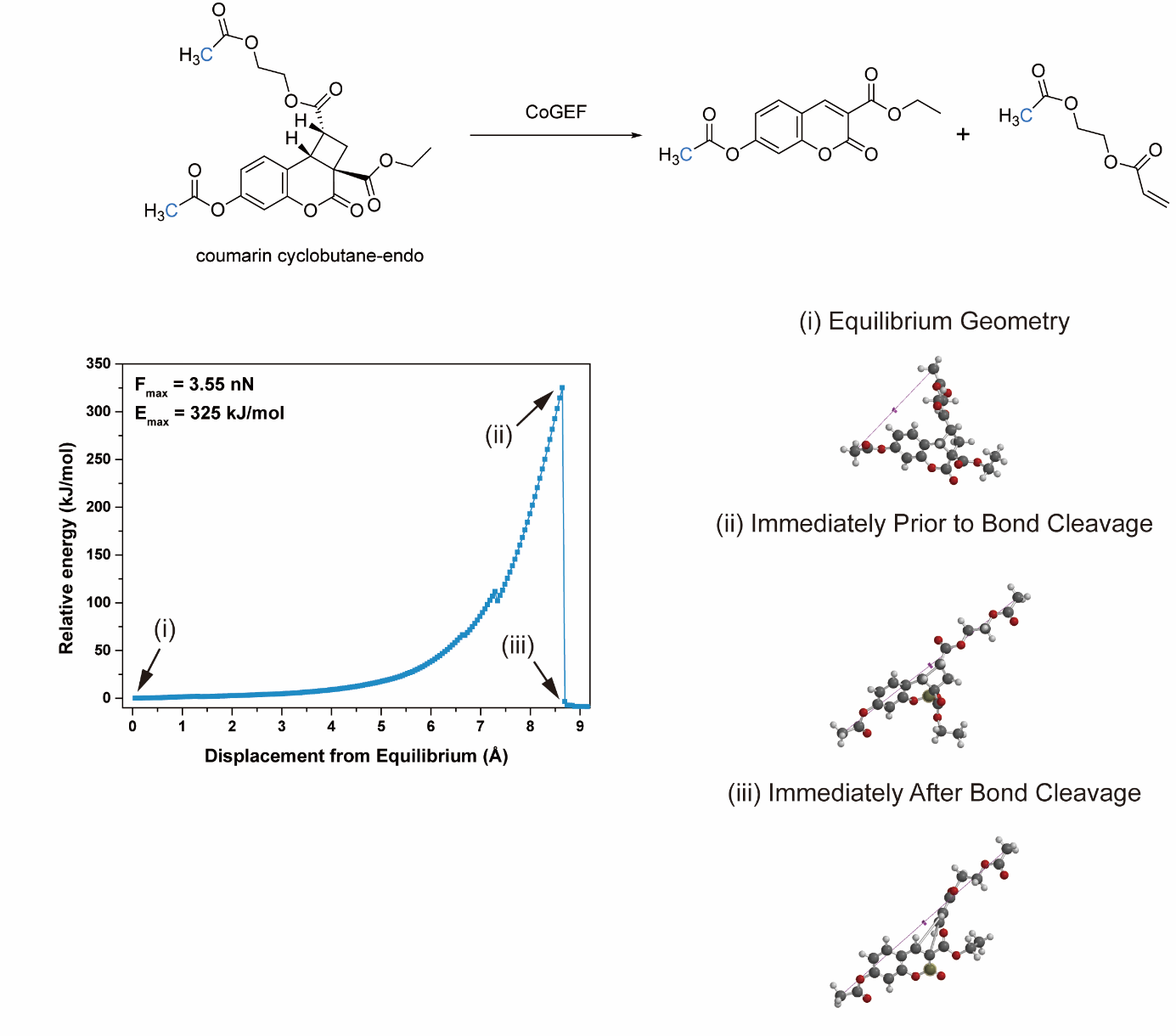


**Figure S1.** DFT calculations using CoGEF at the B3LYP/6-31G* level of theory for the mechanical elongation of the endo coumarin-acrylate adduct, which predict the desired cycloreversion and the activation of coumarin. The atoms colored blue indicate the pulling points for defining the distance constraint.


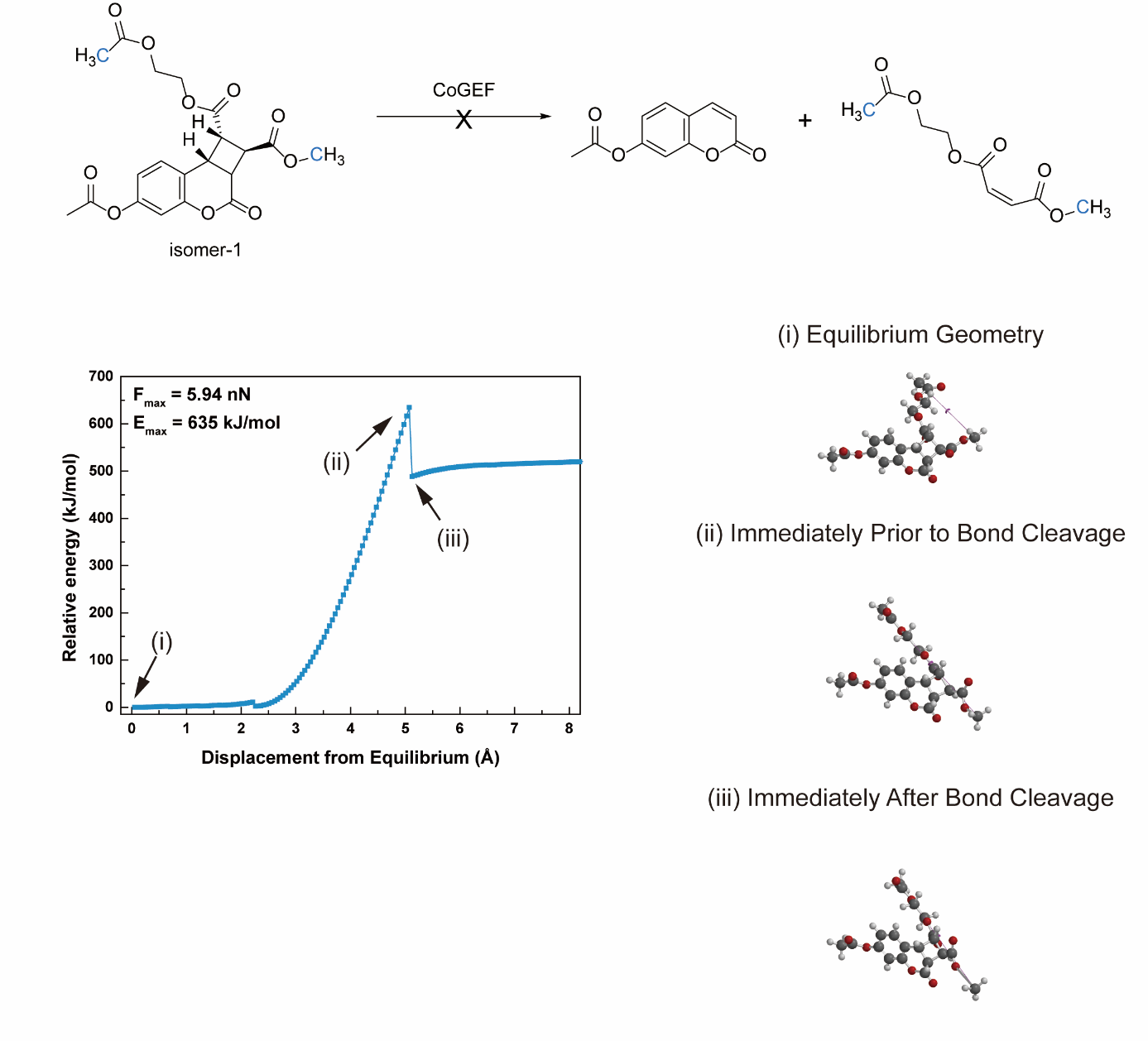


**Figure S2.** DFT calculations using CoGEF at the B3LYP/6-31G* level of theory for the mechanical elongation of a control coumarin-acrylate adduct in which the pulling points are connected to the C1 and C4 positions on the cyclobutane. The cyclobutane is not predicted to undergo cycloreversion. The atoms colored blue indicate the pulling points for defining the distance constraint.


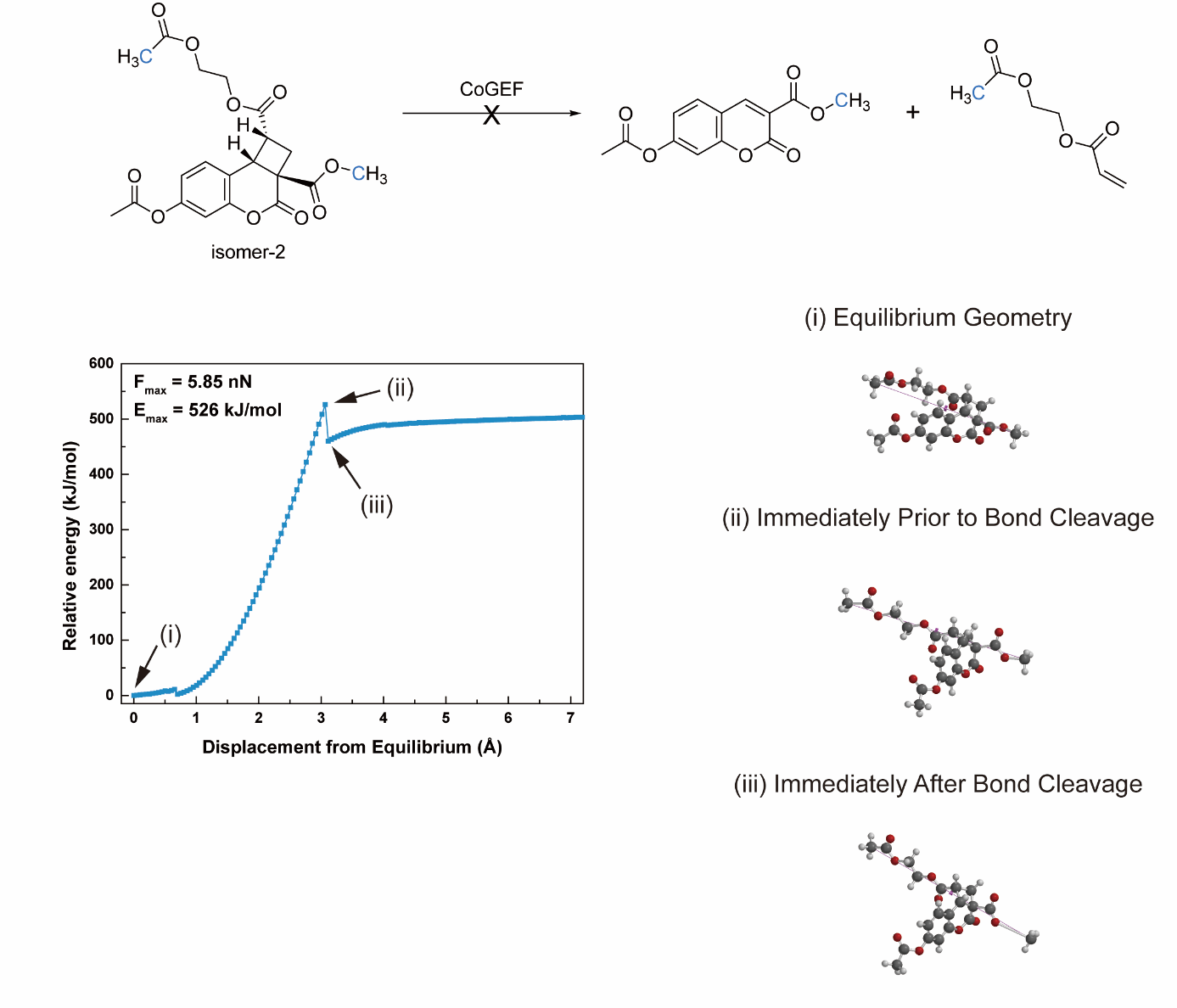


**Figure S3.** DFT calculations using CoGEF at the B3LYP/6-31G* level of theory for the mechanical elongation of a control coumarin-acrylate adduct in which the pulling points are connected to the C1 and C2 positions on the cyclobutane. The adduct is not predicted to undergo cycloreversion. The atoms colored blue indicate the pulling points for defining the distance constraint.


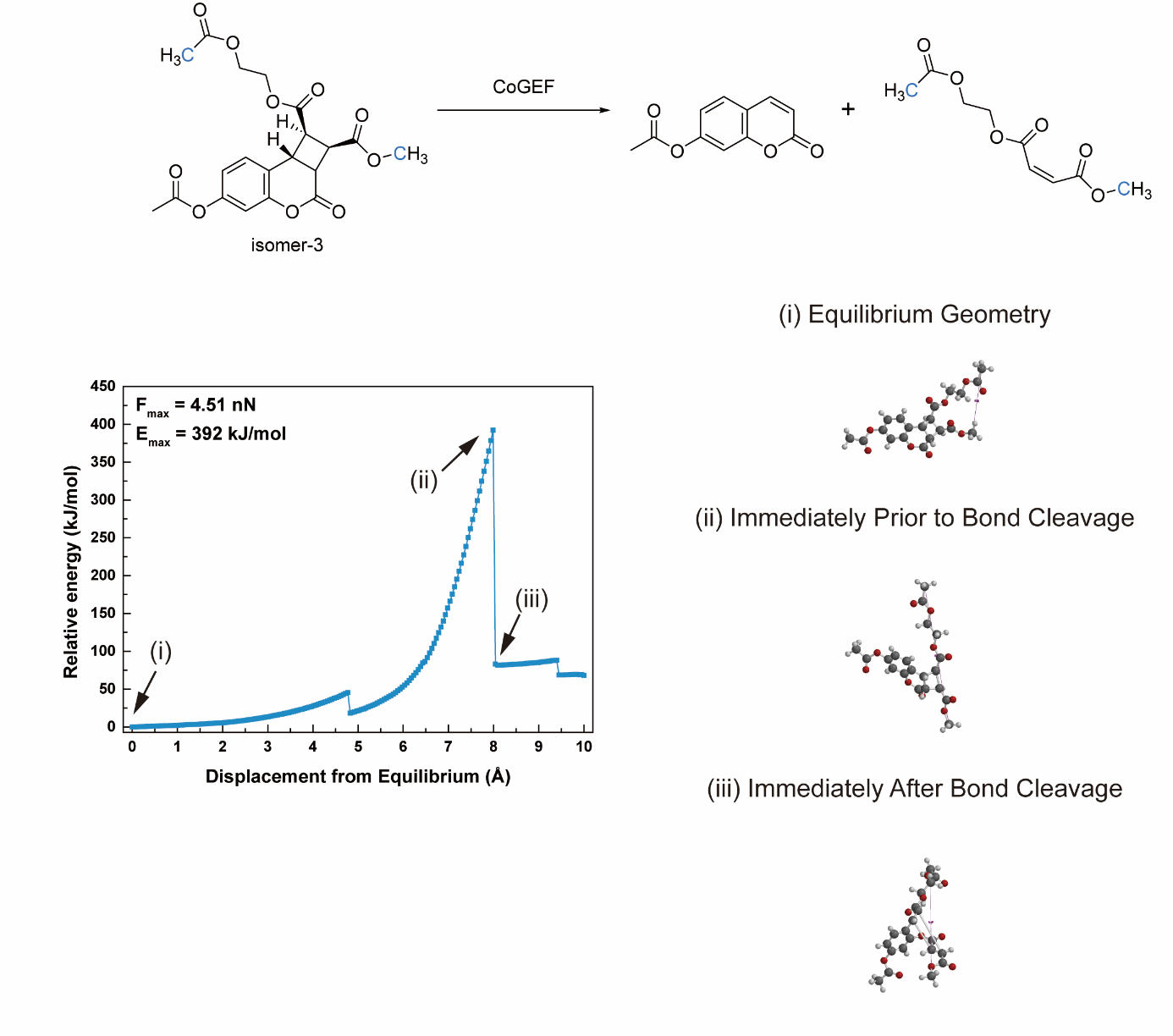


**Figure S4.** DFT calculations using CoGEF at the B3LYP/6-31G* level of theory for the mechanical elongation of a coumarin-acrylate adduct in which the pulling points are connected to the C1 and C4 positions on the cyclobutane. The atoms colored blue indicate the pulling points for defining the distance constraint.


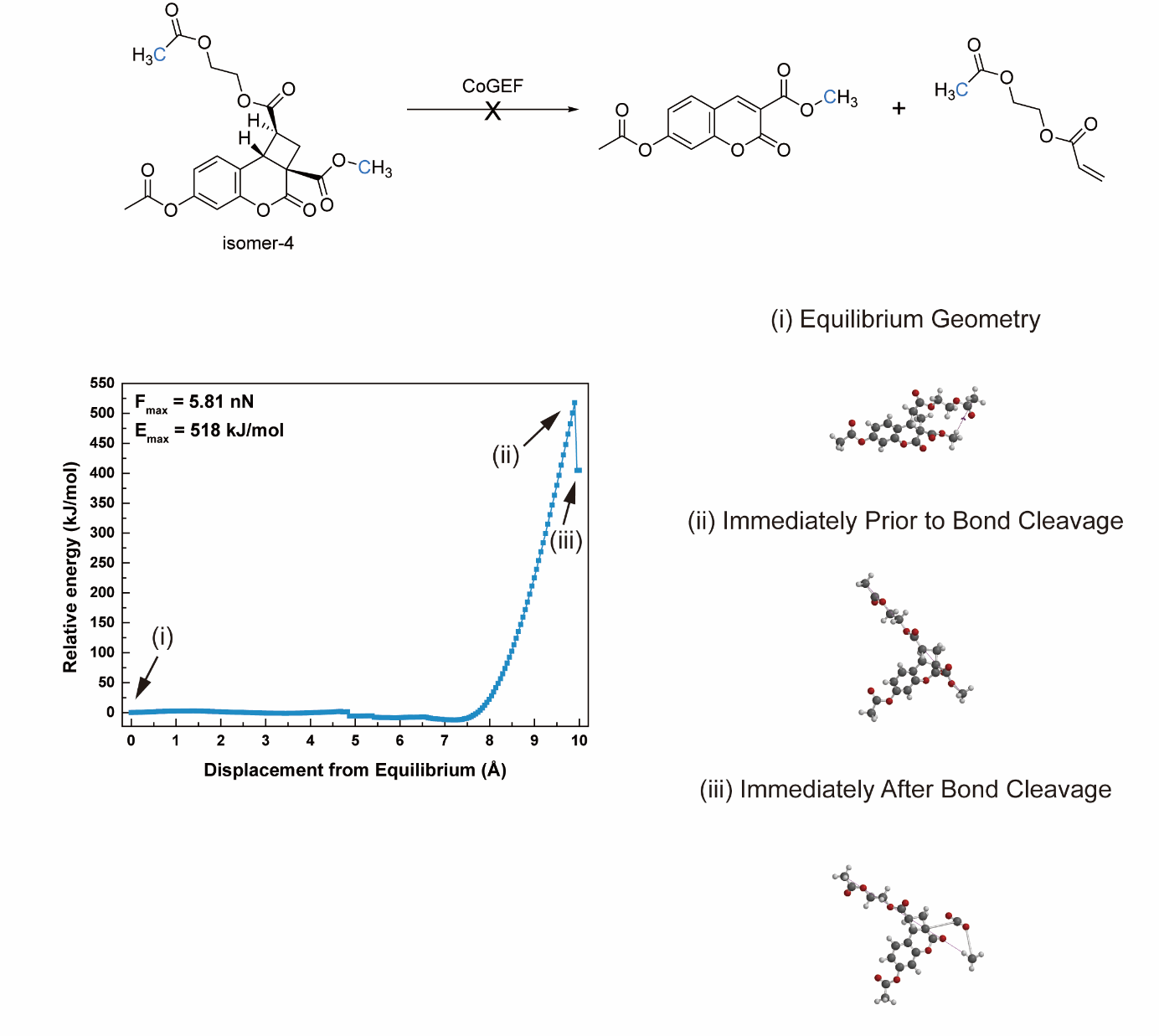


**Figure S5.** DFT calculations using CoGEF at the B3LYP/6-31G* level of theory for the mechanical elongation of a control coumarin-acrylate adduct in which the pulling points are connected to the C1 and C2 positions on the cyclobutane. The adduct is not predicted to undergo cycloreversion. The atoms colored blue indicate the pulling points for defining the distance constraint.


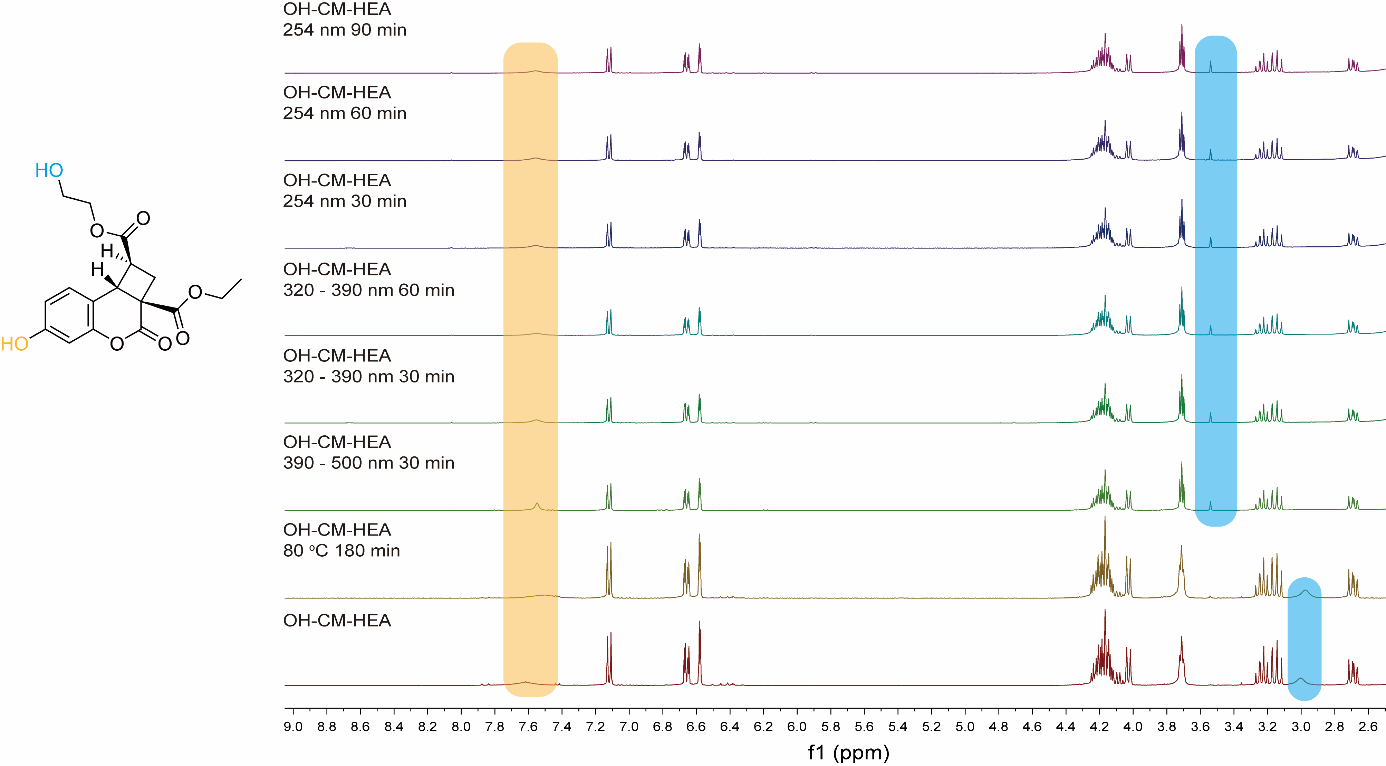


**Figure S6.** ^1^H-NMR spectra show no significant changes in the coumarin-acrylate diol adduct (**OH-CM-HEA**) when subjected to elevated temperatures or to UV or visible light. From bottom to top: **OH-CM-HEA heated** at 80 ^o^C, **OH-CM-HEA** after 30 min 390 – 500 nm irradiation, **OH-CM-HEA** after 30 min 320 – 390 nm irradiation, **OH-CM-HEA** after 60 min 320 – 390 nm irradiation, **OH-CM-HEA** after 30 min 254 nm irradiation, **OH-CM-HEA** after 60 min 254 nm irradiation and **OH-CM-HEA** after 90 min 254 nm irradiation. The orange and blue highlights represent the active hydrogen of the hydroxyl groups in OH-CM-HEA.





**Figure S7.** UV-Vis spectrum of **OH-CM-HEA** in MeCN (concentration 67 μM).





**Figure S8.** TGA curve of **OH-CM-HEA**.





**Figure S9.** DSC curve of **OH-CM-HEA**.


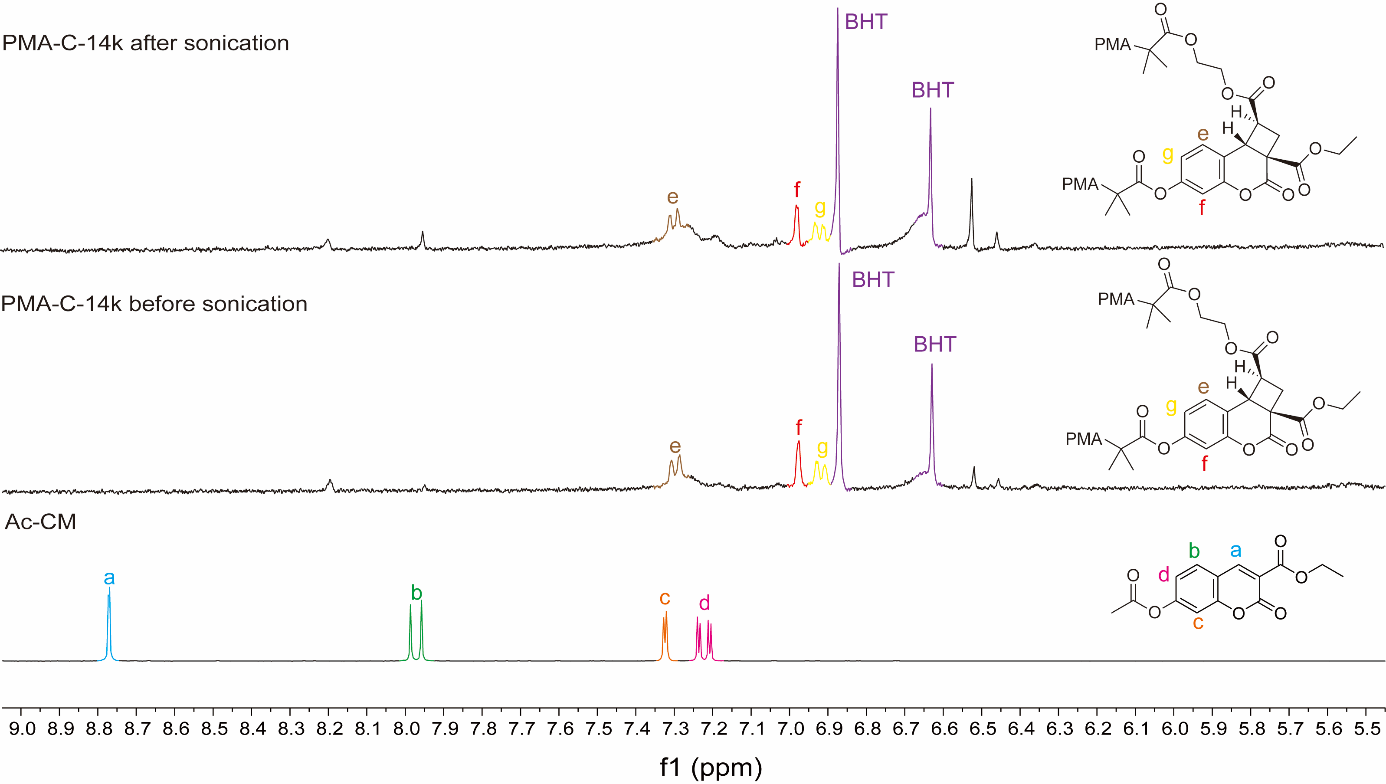


**Figure S10.** ^1^H-NMR spectra show no significant changes in the coumarin-acrylate adduct in low *M*_w_ **PMA-C-14k** subjected to ultrasound sonication. From bottom to top: **Ac-CM**, **PMA-C-14k** before and after 80 min sonication.


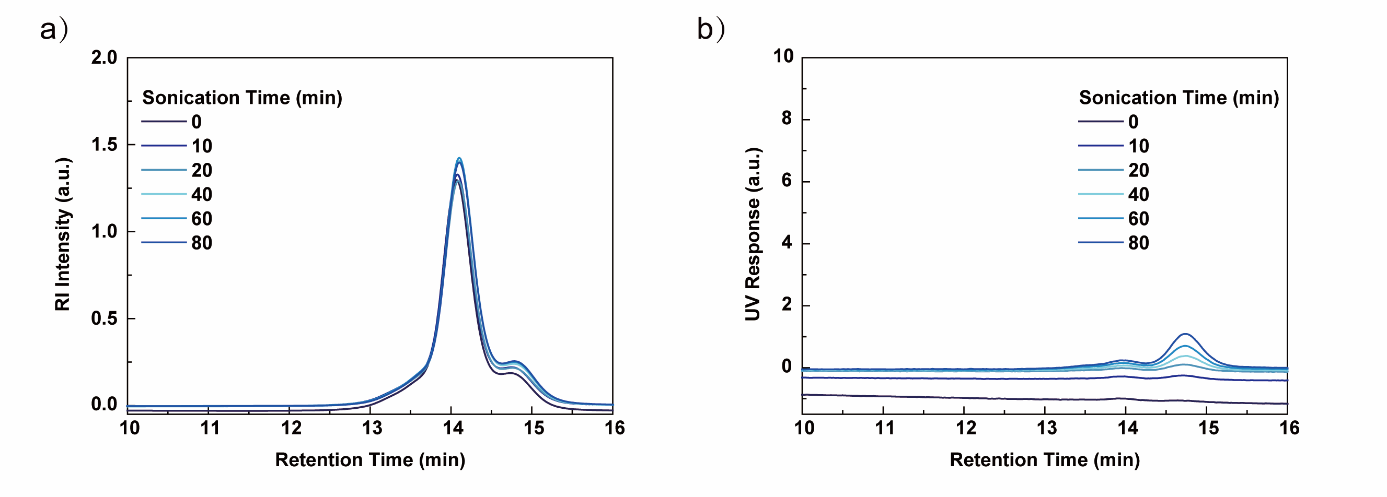


**Figure S11.** (a) SEC-RI traces of **PMA-C-14k** (b) SEC-UV (346 nm) traces of **PMA-C-14k** for different sonication times.


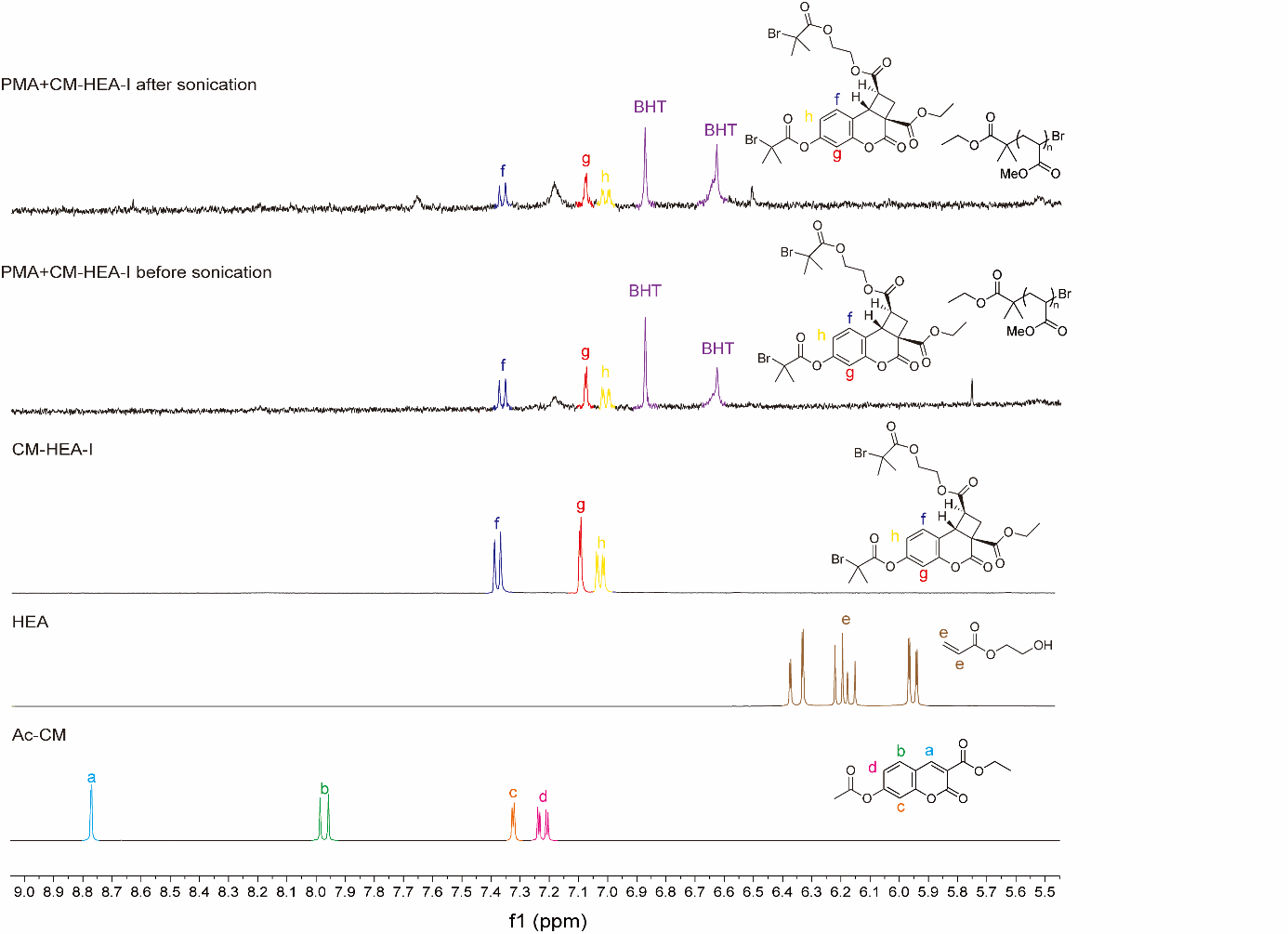


**Figure S12.** ^1^H-NMR spectra show no significant changes in the resonances of **CM-HEA-I** dissolved in acetonitrile (concentration 19.5 μM) with high *M*_w_ **PMA** (150 kDa, 5 mg/mL) following ultrasound sonication. From bottom to top: **Ac-CM**, **HEA**, **CM-HEA-I**, **PMA+CM-HEA-I** before sonication and **PMA+CM-HEA-I** after 80 min sonication.


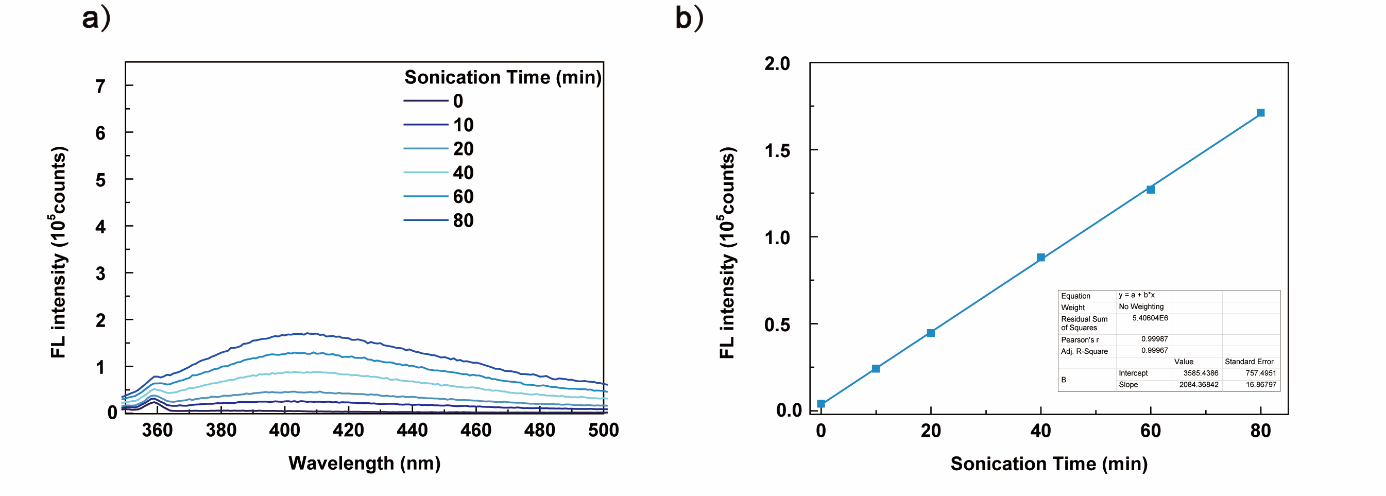


**Figure S13.** Calibration curve construction for background fluorescence spectra upon sonication of acetonitrile. (a) Fluorescence spectra (*λ*_ex_ = 325 nm) of acetonitrile sonicated for different effective sonication times, and (b) Fluorescence emission intensity at 405 nm for pure acetonitrile as a function of effective sonication time. A linear regression applied to the data in (b) gives the calibration function used for background subtraction.


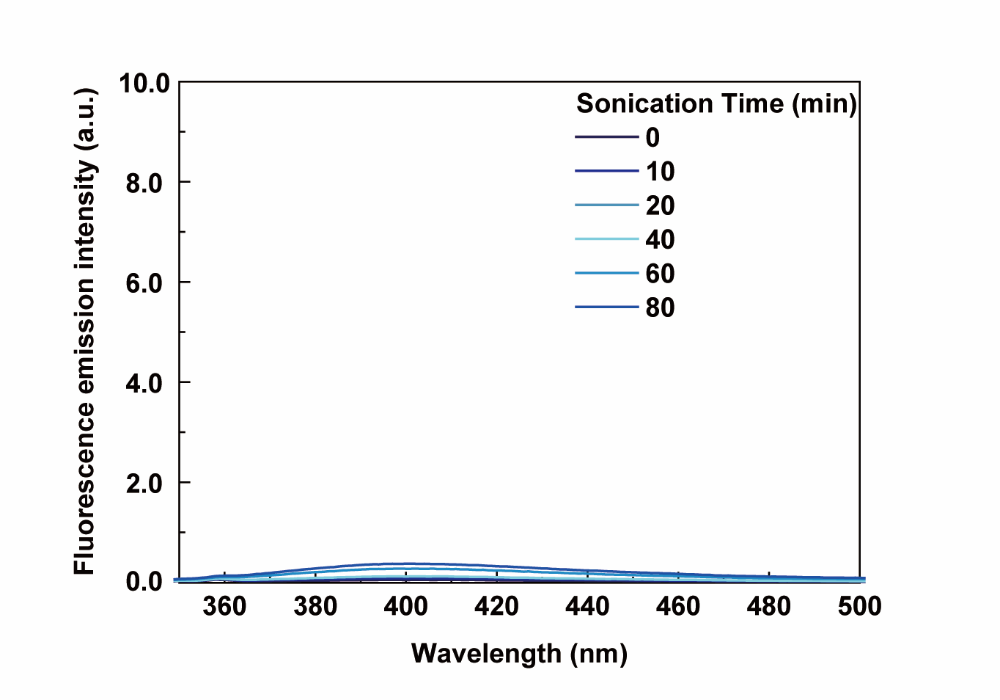


**Figure S14.** Fluorescence spectra of **PMA-C-14k** corrected for background fluorescence following the procedure described in Supplementary Text S4 based on Robb et al.^[1]^ for different effective sonication times.





**Figure S15.** Fluorescence spectra of **CM-HEA-I** before and after 80 minutes of sonication in the presence of in high *M*_w_ **PMA** (150 kDa) in acetonitrile, *λ*_ex_ = 325 nm. The spectra were recorded at a concentration of 9.8 μM CM-HEA-I and 2.5 mg mL^-1^ PMA in acetonitrile, and corrected as described above (Supplementary Text S4).


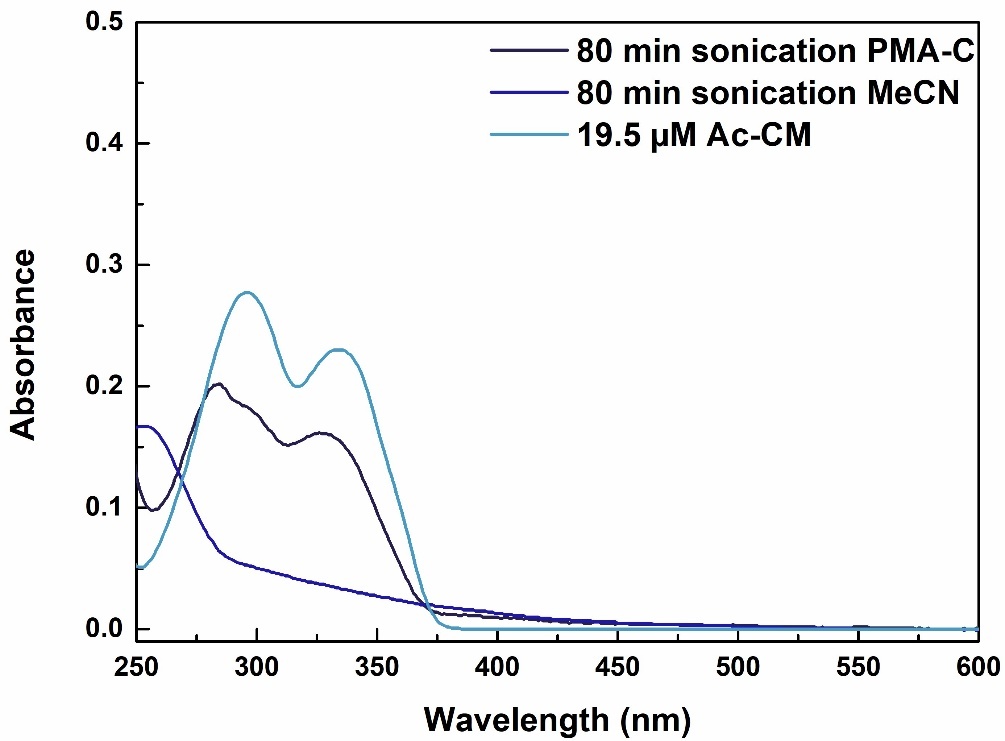


**Figure S16.** UV-Vis spectrum of PMA-C and MeCN after 80 minutes of effective sonication time and **Ac-CM** in MeCN (concentration 19.5 μM).





**Figure S17.** Normalized excitation and fluorescence spectra of **Ac-CM** in MeCN, measured at 325 nm excitation and 400 nm emission (concentration 25 μM).


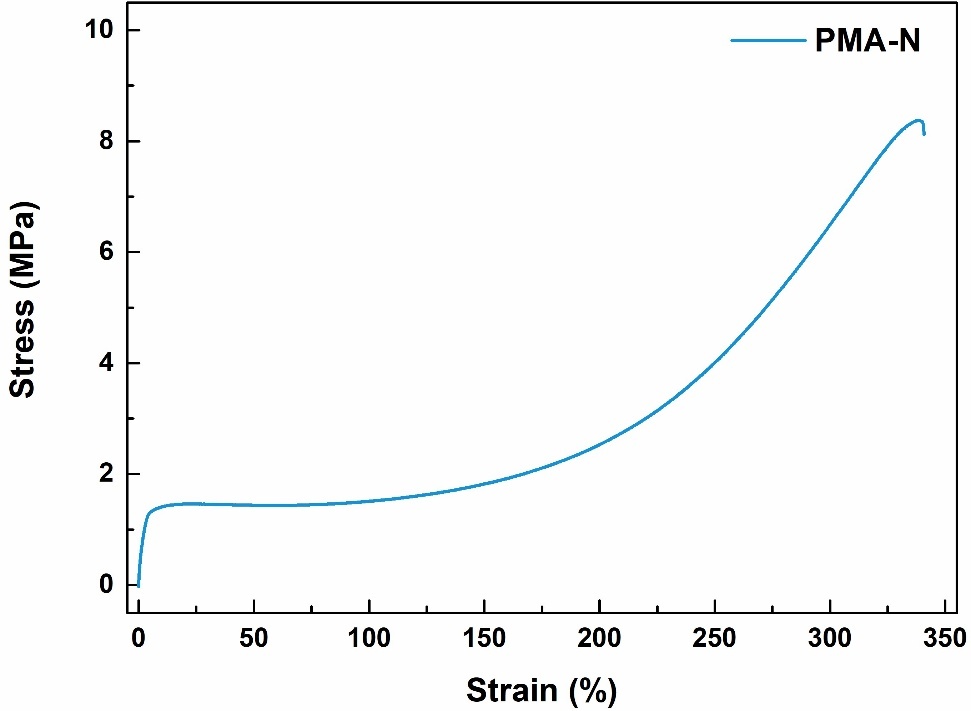


**Figure S18.** Stress–strain curve of **PMA-N**. Tensile test conducted at ambient temperature (22 ^o^C) and at a strain rate of 10.5 % s^-1^.





**Figure S19.** Stress–strain curve of **PMA-PEGDA**. Tensile test conducted at ambient temperature and at a strain rate of 10.5 % s^-1^.

# NMR spectra


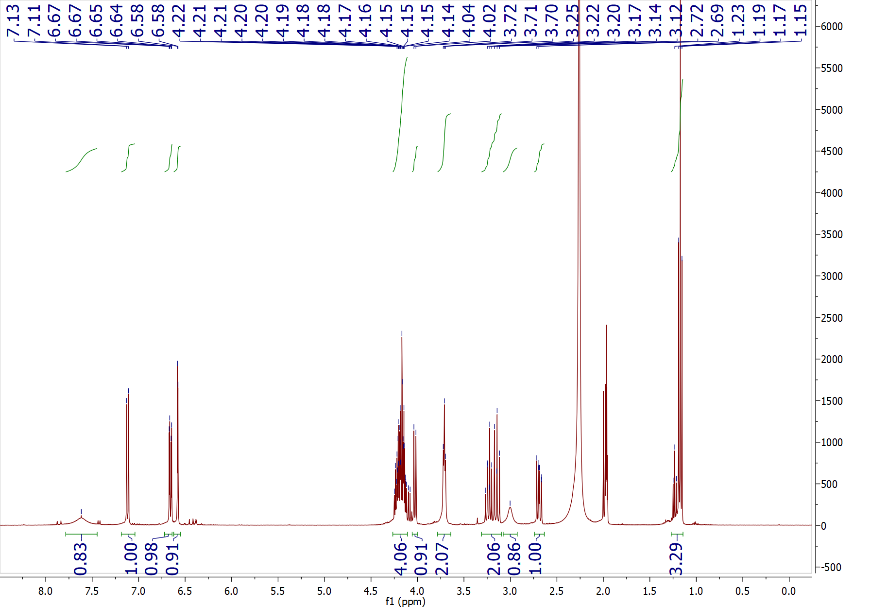


**Figure S20.** ^1^H-NMR (400 MHz in CD_3_CN at 298 K) of **OH-CM-HEA**.


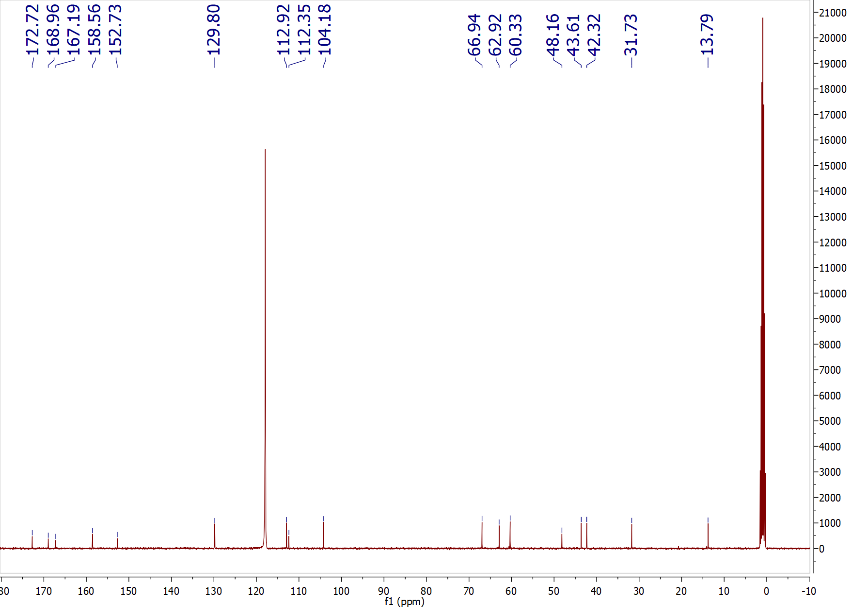


**Figure S21.** ^13^C-NMR (101 MHz in CD_3_CN at 298 K) of **OH-CM-HEA**.


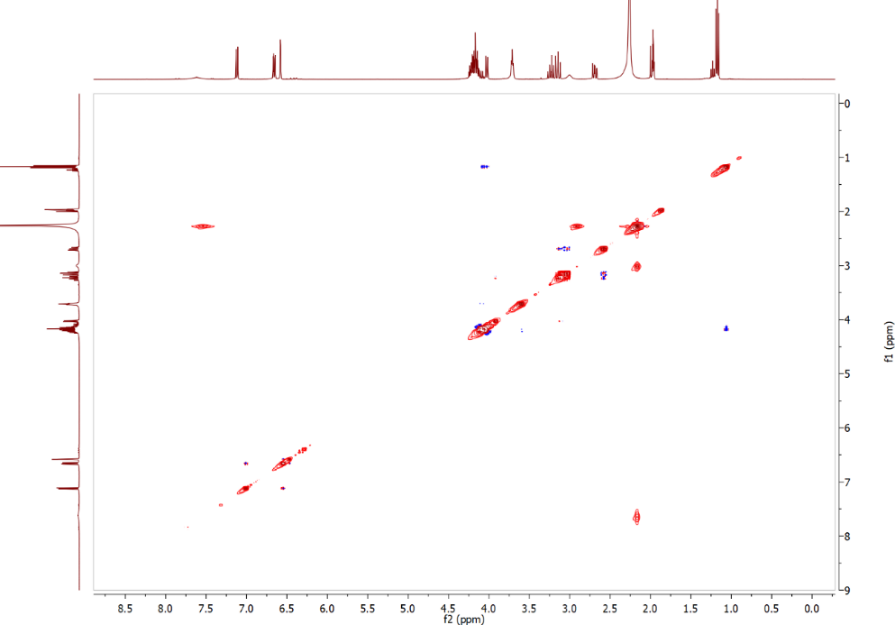


**Figure S22.** 2D-NOESY spectra (400 MHz in CD_3_CN at 298 K) of **OH-CM-HEA**.


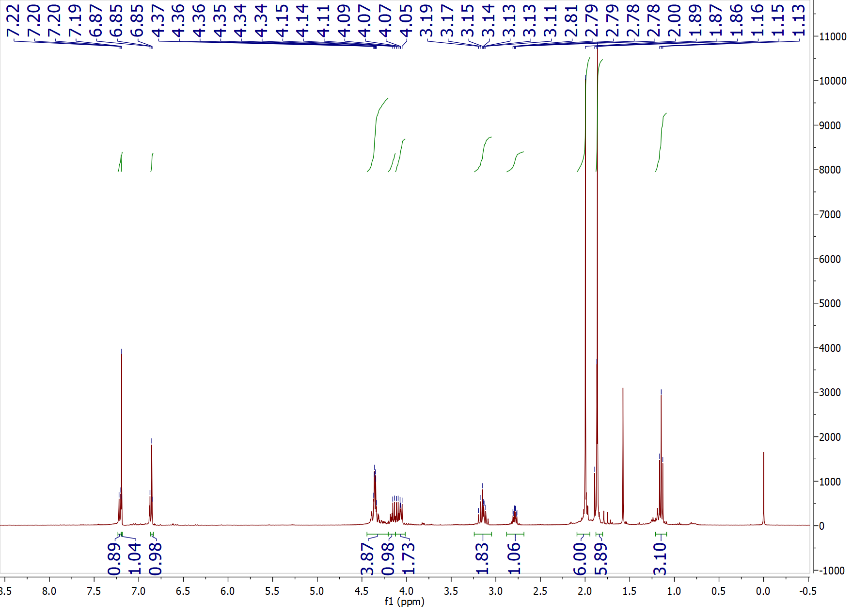


**Figure S23.** ^1^H-NMR (400 MHz in CDCl_3_ at 298 K) of **CM-HEA-I**.


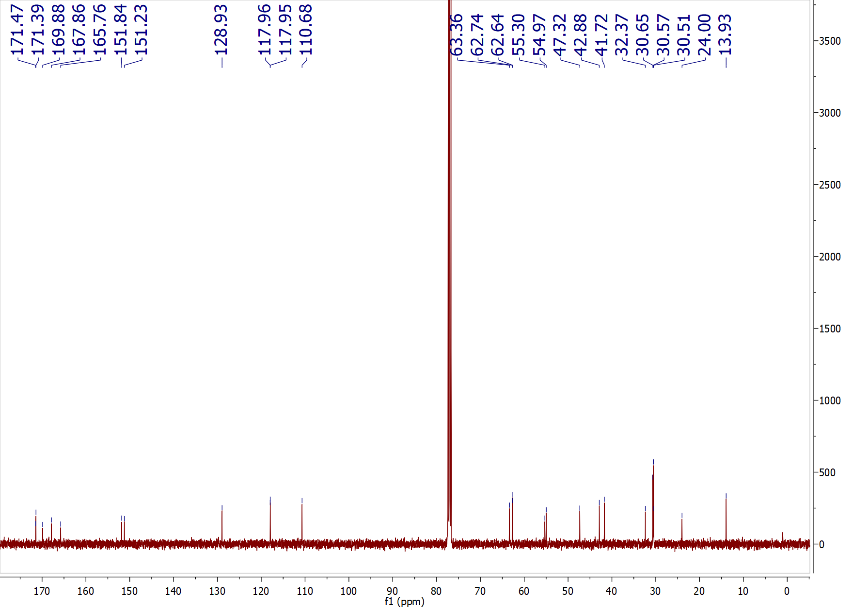


**Figure S24.** ^13^C-NMR (101 MHz in CDCl_3_ at 298 K) of **CM-HEA-I**.


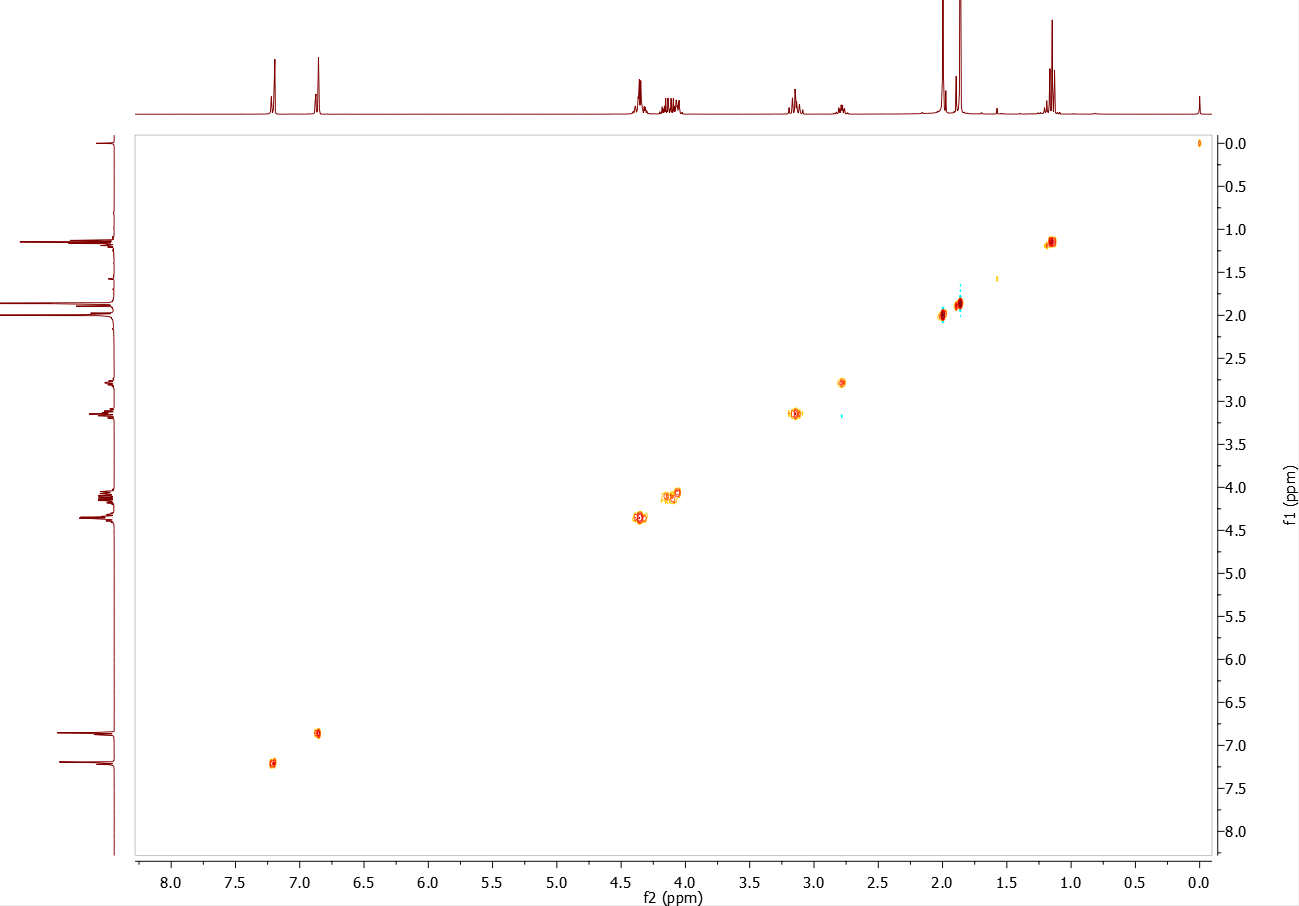


**Figure S25.** 2D-NOESY spectra (400 MHz in CDCl_3_ at 298 K) of **CM-HEA-I**.


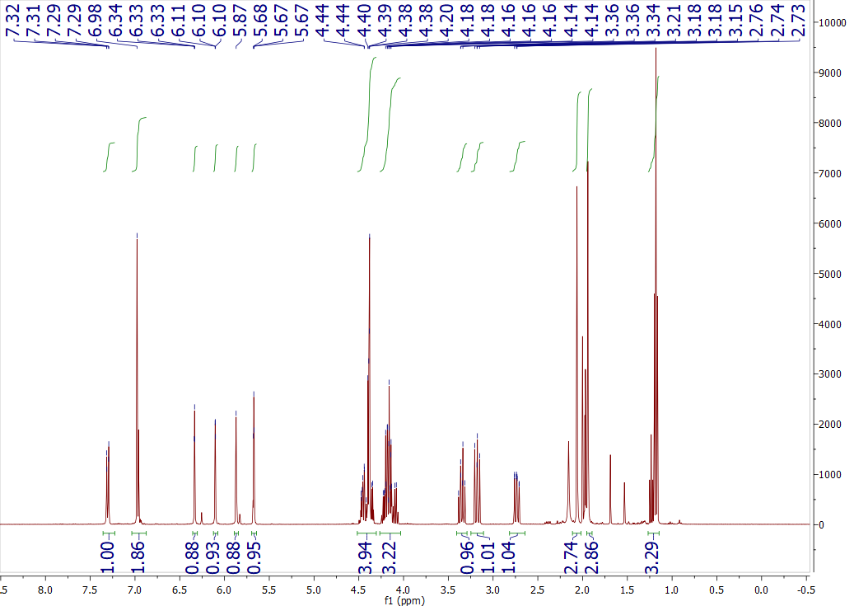


**Figure S26.** ^1^H-NMR (400 MHz in CD_3_CN at 298 K) of **CM-HEA-X**.


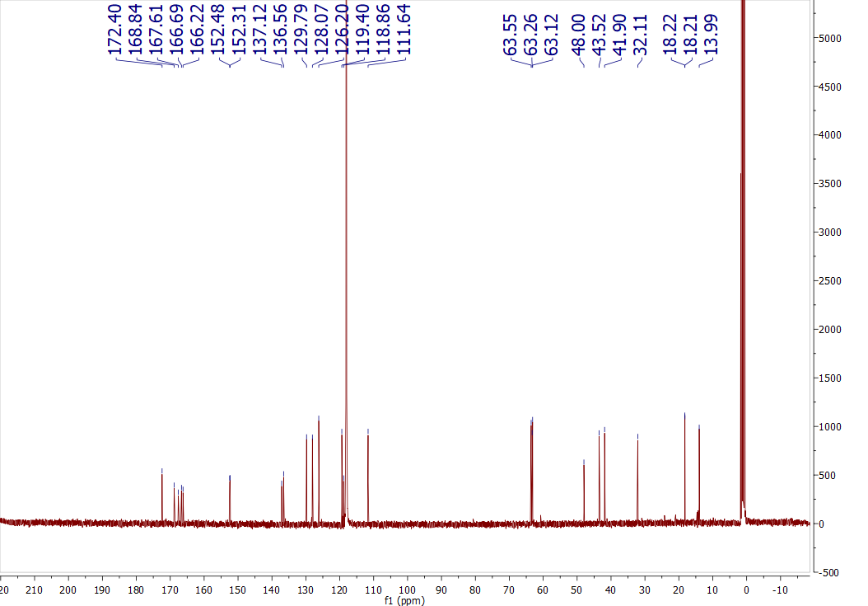


**Figure S27.** ^13^C-NMR (101 MHz in CD_3_CN at 298 K) of **CM-HEA-X**.


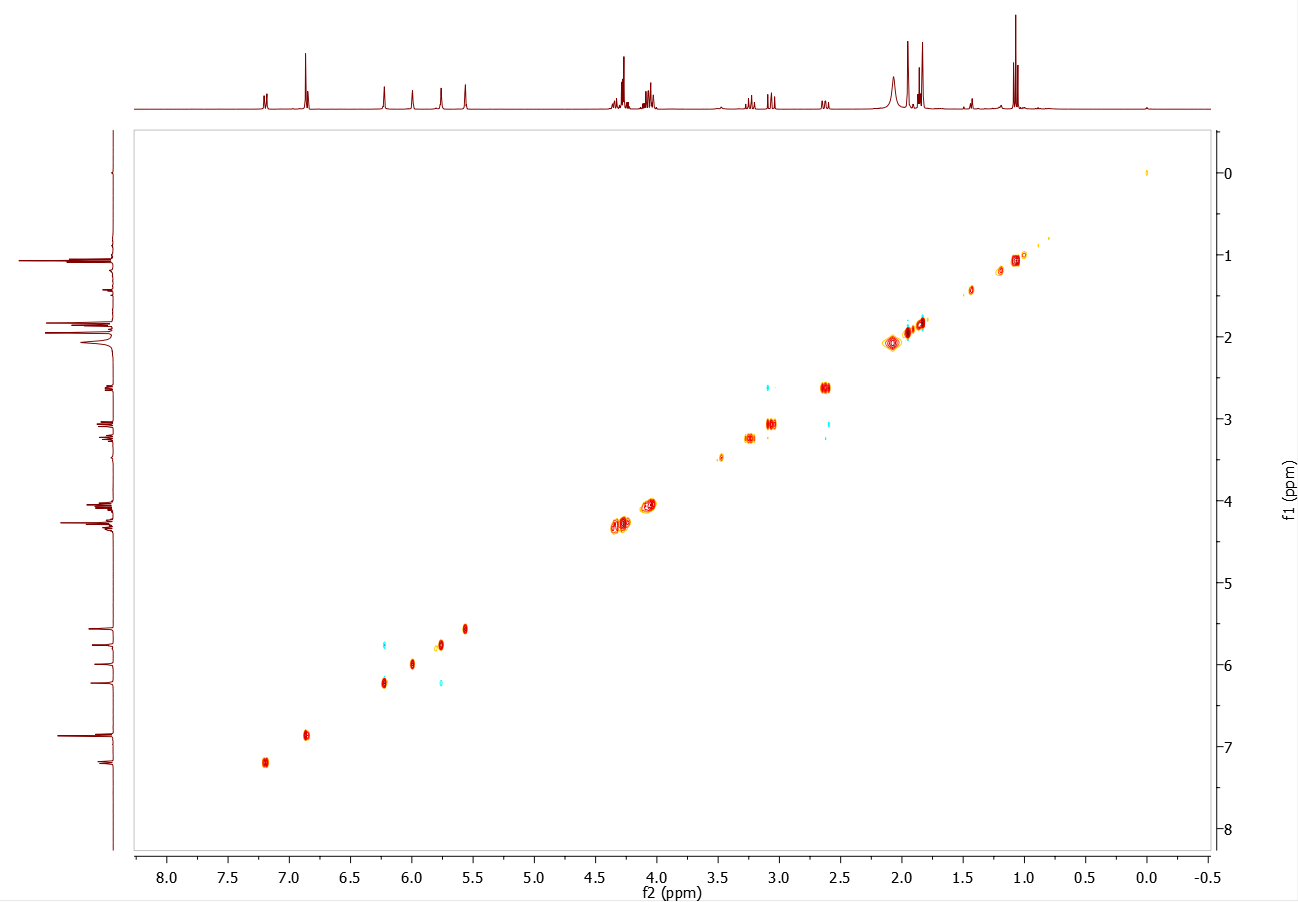


**Figure S28.** 2D-NOESY spectra (400 MHz in CD_3_CN at 298 K) of **CM-HEA-X**.


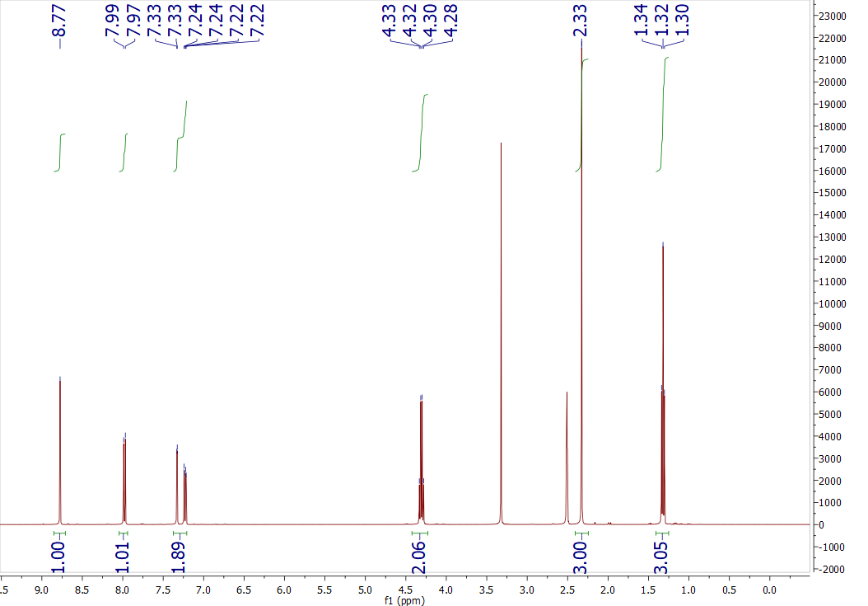


**Figure S29.** ^1^H-NMR (400 MHz in DMSO-d_6_ at 298 K) of **Ac-CM**.


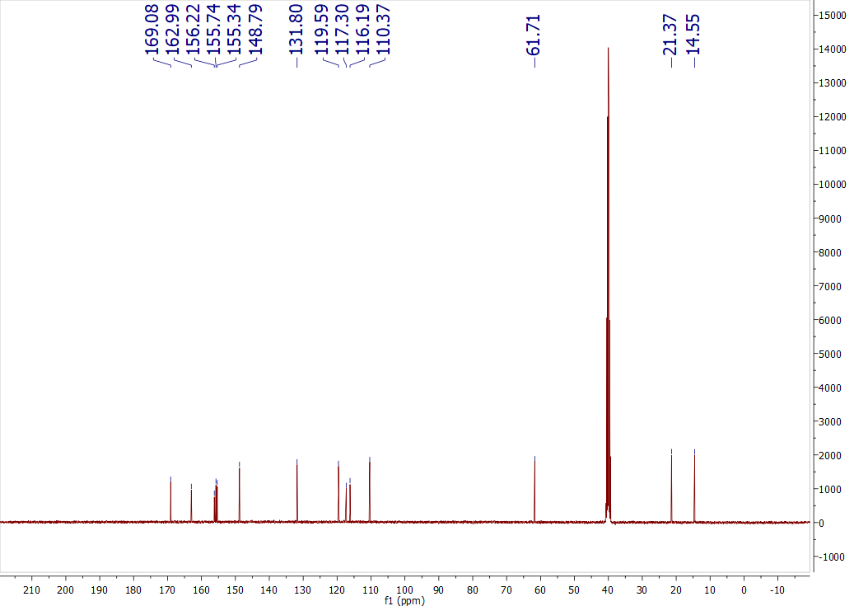


**Figure S30.** ^13^C-NMR (101 MHz in DMSO-d_6_ at 298 K) of **Ac-CM**.


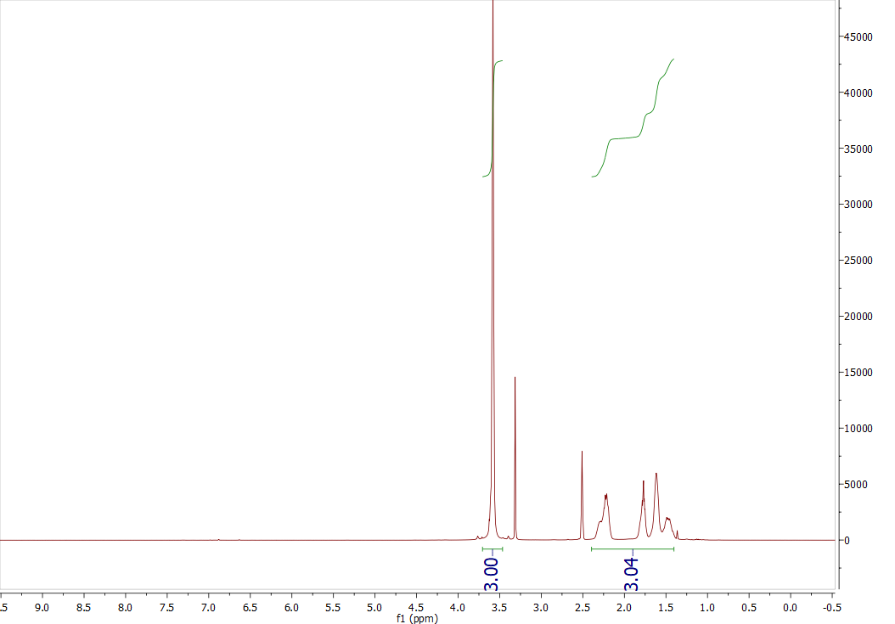


**Figure S31.** ^1^H-NMR (400 MHz in DMSO-d_6_ at 298 K) of **PMA-C-128k**.


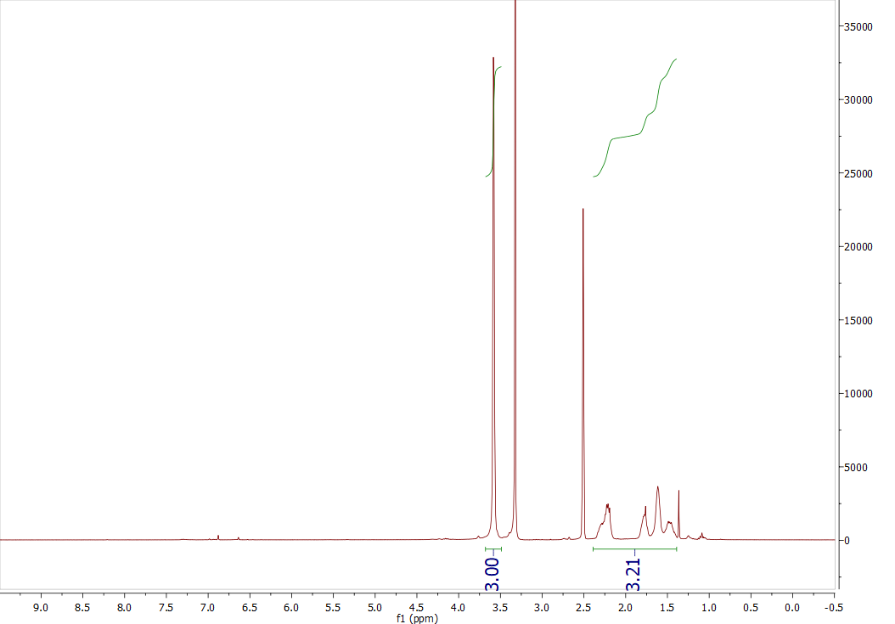


**Figure S32.** ^1^H-NMR (400 MHz in DMSO-d_6_ at 298 K) of **PMA-C-14k**.

# References

[1] I. M. Klein, C. C. Husic, D. P. Kovács, N. J. Choquette, M. J. Robb, *J. Am. Chem. Soc.* **2020**, *142*, 16364–16381.

[2] M. K. Beyer, *J. Chem. Phys.* **2000**, *112*, 7307–7312.

[3] Q. Liu, F. P. Zhu, X. L. Jin, X. J. Wang, H. Chen, L. Z. Wu, *Chem. - A Eur. J.* **2015**, *21*, 10326–10329.

[4] F. Yang, T. Geng, H. Shen, Y. Kou, G. Xiao, B. Zou, Y. Chen, *Angew. Chemie - Int. Ed.* **2023**, *62*, e202308662.

[5] W. J. Liao, S. Y. Lin, Y. S. Kuo, C. F. Liang, *Org. Lett.* **2022**, *24*, 4207–4211.

[6] H. Traeger, Y. Sagara, D. J. Kiebala, S. Schrettl, C. Weder, *Angew. Chemie - Int. Ed.* **2021**, *60*, 16191–16199.

[7] N. Willis-Fox, E. Rognin, C. Baumann, T. A. Aljohani, R. Göstl, R. Daly, *Adv. Funct. Mater.* **2020**, *30*, 2002372.

[8] A. C. Overholts, M. J. Robb, *ACS Macro Lett.* **2022**, *11*, 733–738.

[9] S. Aydonat, D. Campagna, S. Kumar, S. Storch, T. Neudecker, R. Göstl, *J. Am. Chem. Soc.* **2024**, *146*, 32117–32123.

[10] S. M. Luo, R. W. Barber, A. C. Overholts, M. J. Robb, *ACS Polym. Au* **2023**, *3*, 202–208.
